# Navigating direct-to-consumer genetic testing: experiences, decisions and perspectives of Dutch users

**DOI:** 10.1038/s41431-026-02022-z

**Published:** 2026-02-04

**Authors:** Danny Bruins, Esther A. M. Bührman, Martina C. Cornel, Marc H. W. van Mil, Margreet G. E. M. Ausems, Olga C. Damman, Tessel Rigter

**Affiliations:** 1 Section Community Genetics, Department of Human Genetics, Amsterdam Public Health Research Institute, Personalized Medicine, Amsterdam UMC, Vrije Universiteit Amsterdam, Amsterdam, The Netherlands; 2 Center of Education and Training, University Medical Center Utrecht, Utrecht, The Netherlands; 3 Center for Molecular Medicine, University Medical Center Utrecht, Utrecht, The Netherlands; 4 Department of Genetics, Division Laboratories, Pharmacy and Biomedical Genetics, University Medical Center Utrecht, Utrecht, The Netherlands; 5 Department of Public and Occupational Health, Amsterdam Public Health Research Institute, Quality of Care, Amsterdam UMC, Vrije Universiteit Amsterdam, Amsterdam, the Netherlands; 6 Center for Health Protection, National Institute for Public Health and the Environment, Bilthoven, The Netherlands

**Keywords:** Public health, Medical genetics, Genetics, Genetics research, Ethics

## Abstract

Insights into the perspectives, decision-making and experiences of non-US consumers regarding health-related direct-to-consumer genetic testing (DTC-GT) are currently lacking. These insights are essential to allow the implementation of consumer-tailored approaches that facilitate responsible use of DTC-GT. To fill this knowledge gap, the present study employed interviews to examine the consumer journeys of twenty Dutch health-related DTC-GT consumers. Overall, participants appeared quite satisfied with their DTC-GT consumer journeys. Participants’ initial contacts with DTC-GT, as well as their pre-test information acquisition, occurred via a diversity of sources. Participants’ pre-test expectations revealed considerable presumed clinical utility of DTC-GT. Feeling unheard within the regular healthcare system supported multiple participants’ decisions to undergo health-related DTC-GT. Participants mentioned a modifying effect of price on their decision-making, and several participants stated not having considered potential negative consequences of DTC-GT prior to DTC-GT usage. Several potentially adverse consequences of undergoing DTC-GT were identified that could affect individual consumers, the regular healthcare system, and society as a whole. Three considerations to potentially stimulate responsible use of DTC-GT aligning with participants’ needs and preferences were derived, namely improving pre-test information provision, implementing adequate post-test support systems for consumers, and development, implementation and enforcement of cross-border regulation and legislation. Based on these findings, we advocate for stakeholder discussions to further explore the feasibility and desirability of translating these considerations into deliverables. Ultimately, these deliverables could aid in empowering (potential) consumers for responsible use of health-related DTC-GT.

## Introduction

Health-related direct-to-consumer genetic tests (DTC-GTs) are DNA tests sold by commercial companies directly to end users. These tests claim to allow people to gain insight into personal health- and disease risks based on their genetic make-up. Health-related DTC-GTs assess traits such as drug sensitivities, food (in)tolerances, athleticism, and disease risks. Frequently, DTC-GT sellers combine testing for multiple traits within one single test. Oftentimes, there is no qualified healthcare professional (HCP) involved in the DTC-GT consumer journey, which ranges from initial exposure of consumers to the concept of DTC-GT, to the consumer (and other stakeholders) experiencing the impact of DTC-GT usage [1, 2].

Few studies have investigated the perspectives, decision-making and experiences of health-related DTC-GT consumers outside the USA. In recent systematic reviews, the percentage of included articles focusing solely on consumers from the USA ranged from 68% to 90% [3–6]. Since the organization of healthcare systems and healthcare delivery models differ between European countries and the USA, but also within Europe [7, 8], it is likely that perspectives, decision-making, and experiences regarding DTC-GT vary between populations. Indeed, significant variation has been observed between cohorts of DTC-GT consumers from different EU countries regarding perceptions, attitudes, and intended actions in the context of sharing behavior and health-related behaviors [2, 9, 10]. Combined with the potentially severe medical and psychosocial impacts of DTC-GT usage [3, 5], it appears crucial to increase insights into DTC-GT consumers’ perspectives. These insights are essential to enable tailoring of approaches for safeguarding and stimulating responsible use of health-related DTC-GT to specific consumer populations in a way consistent with the population’s preferences, needs, norms and values, also known as societal alignment [11].

For the Netherlands, these insights are lacking, while there is an apparent rise of health-related DTC-GT usage [12–15]. In our study, we utilized an interview-based qualitative approach to study the consumer journeys of Dutch health-related DTC-GT consumers. We wanted to uncover consumers’ perspectives, decision-making and experiences regarding health-related DTC-GT, aiming to ultimately discern considerations in-line with consumers’ preferences and needs in the context of safeguarding and stimulating responsible use of health-related DTC-GT.

## Materials and Methods

### Eligibility criteria & recruitment

Individuals were eligible for inclusion if they had undergone health-related DTC-GT in the past 5 years (either directly via a health-related DTC-GT seller, or via third-party interpretation of raw genetic data obtained from e.g., ancestry-related DTC-GT), had no professional experience in genetics, and spoke Dutch. Thus, relatively recent experiences were collected, while bias towards participants with high knowledge levels was prevented, since professionals in genetics are known to be early adopters of these types of tests [16].

Based on estimates to achieve saturation for descriptive qualitative work [17], we decided to conduct interviews with twenty consumers. Participants were recruited via a research agency (*n* = 6), social and regular media (*n* = 4), active approaching via DTC-GT review websites (*n* = 6), and snowballing (*n* = 4). Participants could participate in-person or online. Written informed consent was obtained from all participants prior to the interview. All interviews were conducted by the first author (DB).

### Interview procedure

After a short verbal explanation of the study, participants had the opportunity to ask questions. The interviews were structured in such a way that participants implicitly and explicitly reflected on their DTC-GT consumer journeys in a chronological manner [2].

The first interview questions concerned the DTC-GT itself, and the time period before taking the test. Participants were asked to think back specifically to that time period. Firstly, general questions were asked about which health-related DTC-GT participants had done and how long ago. Subsequently, participants’ initial contact with and opinions concerning health-related DTC-GT were assessed. Next, decision-making was analyzed by querying pre-test information acquisition, discussion about potentially undergoing DTC-GT with others, and key arguments driving participants’ decision-making. Thereafter, participants were asked about their pre-test expectations of the DTC-GT with regard to (foreseen utility of) the results and impacts. Finally, reflection on feelings and emotions experienced after using the test kit, but prior to receiving the results was solicited.

Then, the interviewer posed questions regarding the time period after receiving the results, and again asked participants to think back to that time period. Participants were asked to reflect on their post-test emotions, perceived understandability and reliability of results they received, undertaken actions after seeing results (e.g. informing family, implementing lifestyle changes, consulting HCPs), and experienced impacts. Moreover, they were asked about differences between pre-test expectations and post-test experiences.

The interviews concluded with participants evaluating their personal consumer journeys. First, participants were asked to rate their experience on a scale of 1–10, elaborate on potential points for improvement regarding the test itself, reflect on whether or not they would do the test again if they could go back in time, and whether or not they would recommend the test to others. Subsequently, participants were asked whether, in hindsight, they missed any support to augment their decision-making process, or whether they could imagine others missing such tools. Then, participants were asked how they would envision such decision-making support tools, and which stakeholder(s) could play a role in developing and implementing them. Finally, participants were asked to summarize their opinion on health-related DTC-GTs, and whether there was anything else they wanted to add. The full, translated interview-guide can be found in Supplementary Materials 1. Based on these reflections, considerations in-line with consumers’ preferences and needs in the context of safeguarding and stimulating responsible use of health-related DTC-GT were drafted by the authors.

### Data analysis

Interview audio recordings were transcribed verbatim, and transcripts were imported to MaxQDA (version 2022) for data analysis.

Braun and Clarke’s 6-stage approach to thematic analysis was used as a guideline for data analysis [18]. Two authors (DB and EAMB) familiarized themselves with the data from five interviews. Initial codes were inductively generated from these five interviews by DB and EAMB, and subsequently consensus was reached on a preliminary codebook. This codebook was then piloted on a randomly selected, previously unseen interview. The codebook was then finalized (Supplementary Materials 2) following a post-pilot meeting by DB and EAMB, during which discrepancies in coding were resolved until consensus was reached. Following codebook finalization, the entire corpus was deductively coded by DB. Themes were identified and subsequently discussed with all other authors prior to finalization.

## Results

### Interview length & participant demographics

Twenty semi-structured interviews were conducted. Eleven were in-person interviews, nine were online. Interviews lasted between 46 and 121 minutes. Socio-demographic characteristics are shown in Table 1 (with references [19–21]).

**Table 1:** Demographic characteristics of study participants.

Characteristics	Respondents (*n* = 20) *N* (%)
**Age (years)**	
18–39	3 (15)
40–59	11 (55)
≥ 60	6 (30)
**Gender**	
Female	13 (65)
Male	7 (35)
**Education level**	
Low^a^	2 (10)
Intermediate^b^	5 (25)
High^c^	13 (65)
**Religion**	
None	17 (85)
Islam	1 (5)
Catholic	1 (5)
Buddhist	1 (5)
**Ethnic background**	
Dutch, Dutch parents	18 (90)
Moroccan, Moroccan parents	1 (5)
Indonesian, Indonesian father, Dutch mother	1 (5)
**Province of inhabitance**	
North-Holland	7 (35)
South-Holland	5 (25)
North-Brabant	1 (5)
Limburg	2 (10)
Gelderland	2 (10)
Overijssel	2 (10)
Friesland	1 (5)
**Time since undergoing DTC-GT**	
< 1y	9 (45)
1–5y	10 (50)
5–10y	1* (5)
**Health literacy (HL)**^**d**^	
High	19 (95)
Low	1 (5)
**Numeracy**^**e**^	
High	11 (55)
Low	9 (45)

^a^primary school, lower level of secondary school, lower vocational training. ^b^higher level of secondary school, intermediate vocational training ^c^higher vocational training, university ^d^Measured using participants’ responses to validated Dutch translation of ‘Confident with Forms’ question originally defined by Chew et al. 2008. Participants selecting responses ‘helemaal niet zeker’, ‘een klein beetje zeker’, or ‘een beetje zeker’ were classified as having low health literacy (Chew et al., 2008; Fransen et al., 2011). ^e^Measured using Dutch translation of the single-item (median) Berlin Numeracy Test, which estimates those that answer correct as being in the top half of participants in terms of numeracy (designated as ‘high’), whereas those that answer incorrectly belong to the bottom half (designated as ‘low’) (Cokely et al. 2012). *One of the participants stated during the interview to have undergone an ancestry-related DTC-GT 1–5 y ago, and a health-related DTC-GT approximately 10 y ago. Participant thus technically fell outside of inclusion criteria (undergoing health-related DTC-GT ≤ 5 y ago), but since interview was already ongoing when this was uncovered, it was decided to include this participant in the study cohort.

### Categories & themes

**Fig. 1:**
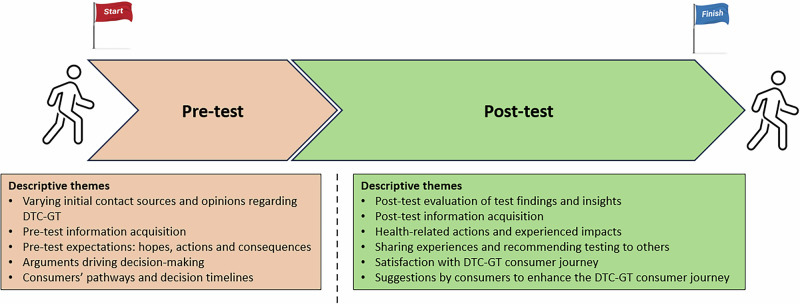
Schematic display of identified themes along the two phases of the DTC-GT consumer journey. Five descriptive themes were identified in the pre-test phase of the DTC-GT consumer journey, and six descriptive themes were identified in the post-test phase.

Eleven descriptive themes were identified and categorized along the two chronological phases in the consumer journey: [1] Pre-test (five themes), encompassing the consumer journey from its start up until the timepoint where a consumer has ultimately decided to undergo health-related DTC-GT and the route through which they will do so, and [2] Post-test (six themes), covering the consumer journey from consumers actually undergoing health-related DTC-GT and onwards (Fig. 1). An extensive table with quotes supporting the themes can be found in Supplementary Materials 3, with quotes from this table referenced in-text where relevant. Additionally, a condensed table with representative quotes per theme can be found in Table 2.

**Table 2:** Selected representative quotes per theme^a^.

Theme	Quote	Quote Number
Varying initial contact sources and opinions regarding DTC-GT	*‘Well, I went digging in my own medical history, and yeah, via Facebook I ended up in certain groups, and via there I got into contact with someone that had also done a DTC-GT. They shared their knowledge with me like: ‘It works like this and that and you can get a lot of information out of that.’, so that’s how I ended up there, through their experience.’ (P3, F42, HL* + *, NUM-, l.80)*	Q1
	*‘[When I heard about health-related DTC-GT for the first time it was] eye-opening. I was thinking: ‘Wow, amazing that this is available in the Netherlands!’, I thought that was all American type of stuff, so I was very happy I could do it too!’ (P1, F59, HL* + *, NUM-, l.41-46)*	Q5
Pre-test information acquisition	*‘Well, I also looked at experiences of previous consumers… If more than half of those are negative, there’s always negative ones, but there mainly have to be positive ones. So I looked at that to see whether it’s really reliable. But based on the reviews and explanation on the [seller’s] website and stuff, yeah I had a good feeling about it.’ (P8, F40, HL* + *, NUM-, l.88)*	Q10
Pre-test expectations: hopes, actions and consequences	*‘Uh, yeah, [I expected that] I would get a lot of information that I could use to adapt my lifestyle. Checking if what we’re doing now is good or whether we’re missing certain things, because we’re predisposed to something we’re not aware of. Checking if we had to finetune some stuff.’ (P5, M54, HL* + *, NUM-, l.170-172)*	Q14
	*‘… It wasn’t that I was feeling miserable or something. No, I just thought it was fun and wanted to get to know myself better. One person does that in their heads, others want to get that on paper.’ (P9, M50, HL* + *, NUM-, l.54)*	Q21
	*‘Expectations? I’d rather say hope. I was running into so much lack of understanding of my vague complaints within the regular healthcare system that I hoped to find something that would stop the constant discussion, that’s how you could summarize it.’ (P3, F42, HL* + *, NUM-, l.226)*	Q22
	*‘Well, if you have something very severe, it might be nice to know in advance, but it also might not be nice to know… I think that it could cause some people to lose their minds like: ‘Well, I can’t be saved anymore, so never mind.’.’ (P11, F68, HL* + *, NUM* + *, l.24-26)*	Q30
	*‘Yeah, and I did warn my kids that I was going to do this and that I was doing one that was very good with privacy, but that you never know for sure, so if their DNA ever ended up on a crime scene somewhere, that I couldn’t do anything about it, but my DNA sat somewhere in the system.’ (P6, F61, HL* + *, NUM* + *, l.298)*	Q33
Arguments driving decision-making	*‘Well the price, it was cheap and, uh, the convenience. You’re getting it sent to your home, you don’t have to leave your house.’ (P10, M40, HL* + *, NUM* + *, l.86)*	Q36
	*‘Well, I have a friend that did the DTC-GT first. That friend was very positive about it… And my friend’s partner is a chemist, whom was really impressed by how it worked. And that person is not impressed quickly… That chemist knows his stuff, so he also understands that what’s being sold, is not some nonsense.’ (P1, F54, HL* + *, NUM-, l.14 and l.196-200)*	Q41
	*‘Being brutally honest, I went into this really impulsively and didn’t really read into it at all.’ (P12, F42, HL* + *, NUM* + *, l.128)*	Q50
	*‘Well, the main thing that went around in the back of my head was privacy, like what happens with your data… You’re giving them your data, so to say. They can say that it’s anonymous, but what is ‘anonymous’? I mean, Facebook should be anonymous as well, but you’re still monitored to some degree, so to say. That gave me some doubts.’ (P15, M41, HL* + *, NUM* + *, l.32-34)*	Q53
	*‘But yeah, it’s the costs that are attached to it that make you think like: ‘That’s almost €200!’, you know?’ (P8, F40, HL* + *, NUM-, l.18)*	Q56
Consumers’ pathways and decision timelines	*‘Actually, I think I decided to do it immediately, maybe thought for an hour, but no more than that.’ (P11, F68, HL* + *, NUM* + *, l.42)*	Q66
	*‘No, no, no I did not immediately do it. I really thought about it for a long time. I think like a year or something.’ (P3, F42, HL* + *, NUM-, l.134)*	Q67
Post-test evaluation of test findings and insights	*‘Well, if you open the report, the most impactful things are listed at the top, and if it’s green, it’s positive, and if something’s negative it’s red.’ (P12, F42, HL* + *, NUM* + *, l.236)*	Q69
	*‘…I had not anticipated the amount of work it would be to be able to understand it [the results]… I think I’ve spent a year puzzling with the results before I understood how to approach it.’ (P6, F61, HL* + *, NUM* + *, l.460)*	Q74
	*‘Well, it’s easy to read. It isn’t explained in layman terms, but it’s also not medicalese… No but look, it’s neat isn’t it? I think that, of course you’re looking at it from your own point of view, but I feel like the average Dutch person should be able to read this right? I feel that this is accessible for the average Dutch person.’ (P1, F59, HL* + *, NUM-, l.694-700)*	Q76
	*‘Yeah, it seemed reliable… because I recognized myself in certain results. It made the results make sense. I would’ve had doubts if they [the results] would’ve been very different.’ (P15, M41, HL* + *, NUM* + *, l.254-258)*	Q79
	*‘I’m taking it with a large grain of salt… because you don’t just have those diseases, but also here for example: ‘80% chance that you have no to little hair on your back’, well, my partner sometimes calls me a silverback gorilla, so yeah [laughs], I’ve got more hair on my back than on my head… ‘77% chance you’ve never had dandruff’, well I did have that. When you look at stuff like that, it makes you think it’s still a bit of guesswork… All those traits, if I look at those, most of which are only so-so correct, how serious do I have to take that medical test then?’ (P2, M70, HL* + *, NUM* + *, l.128-160)*	Q84
Post-test information acquisition	*‘Via PubMed I ended up at that pharmacist, he put me into contact with someone else, who put me into contact with yet someone else, who brought me into contact with the professor. So all of that goes via a single publication on PubMed.’ (P1, F59, HL* + *, NUM-, l.760)*	Q88
	*‘…If you do the comprehensive DTC-GT, you only get half of the information after consultation with an expert of the company, they don’t give you that information without any explanation. We got that information and then they’ll dive even deeper into the processes and abnormalities… That specialist quickly asked ‘Aren’t you suffering from headaches?’ I said ‘No’, but by that time it was so regular for me to suffer from headaches, so I said ‘Oh, yeah, yeah’, and he said: ‘Yeah, because I can see here very clearly, that’s so remarkable, that you process histamine very badly, so large chance that that’ll make you suffer from headaches.’ So he said ‘that’s due to this and that, you’re missing that enzyme, if you supplement that in supplement form, you should notice that’. Well, that was the case.’ (P5, M54, HL* + *, NUM-, l.214)*	Q91
	*‘…So then I went searching for all sorts of genes involved in muscle disorders… and put all of those genes in that program that I just showed you, and all of a sudden one popped up in which I had two mutations that were not OK… So when I found those two mutations, I told my cardiologist, and he said: ‘It is important that we look into that a bit more’, and referred me to the clinical genetics center. There they said: ‘Yeah, two mutations…’, and one was proper bad, that was known, but the other was unknown, but it could very well be bad, so to say, it was located in a not-unimportant part of the gene… So yeah, there’s a large chance that my muscle problems are due to this gene… The conclusion was that you can’t be sure, but I was advised to be careful with it.’ (P6, F61, HL* + *, NUM* + *, l.470-516)*	Q97
	*‘Initially, the oncologist wouldn’t go along with it [the DTC-GT results for drug sensitivity]. He said: ‘That’s nice and all, but we’re not going to do that. The standard dosage is 20 milligrams, so you’re getting 20 milligrams, now get out.’… Eventually I ended up at another hospital, and they gave me 30 milligrams after some measurements.’ (P1, F59, HL* + *, NUM-, l.112-128)*	Q98
Health-related actions and experienced impacts	*‘Well, due to those supplements, there was one that I’d never heard of before, and it turns out from my DNA that I have very high stress levels, which I wasn’t even aware of myself… The therapist got that out of my DNA and I got a certain supplement… which made me feel an awful lot better.’ (P18, F72, HL* + *, NUM-, l.180)*	Q103
	*‘…What I did do at the time of the COVID-19 pandemic, is check if I was at high risk of getting very sick from that, to decide whether or not I should get vaccinated… My mother and I decided not to get vaccinated. No, we were already having our doubts at the time and then the DTC-GT showed that we were at low risk of getting very ill from it… So we took a gamble and didn’t get vaccinated.’ (P14, F38, HL* + *, NUM-, l.18 and l.76)*	Q109
	*‘The BRCA1- and BRCA2-genes, so breast cancer genes, have also been tested, and well, since I’ve hit that jackpot [breast cancer] two times, maybe I’ve got that gene. But I don’t, so that’s a relief. Not that it matters, but it’s nice to know… It’s just pure bad luck.’ (P1, F59, HL* + *, NUM-, l.100-104)*	Q110
	*‘I mean, it’s not a walk in the park, and when I discovered that increased Alzheimer’s disease risk, I struggled with that for a while… The first time I opened the report, it said very large and loud in the first two blocks ‘Alzheimer’s disease’ in red. So that had me thinking like: ‘Whoops.’.’ (P12, F42, HL* + *, NUM* + *, l.132 and l.288)*	Q112
Sharing experiences and recommending testing to others	*‘Actually, I only told my dad like: ‘Oh you know, that stuff with grandpa, that’s not hereditary for me.’, that’s what I said. That’s actually the only thing I told about it. Well, and I did talk about it at home, but only in passing.’ (P8, F40, HL* + *, NUM-, l.406-408)*	Q115
	*‘Yeah, I definitely shared my enthusiasm with others. For example, at the hair salon, you talk about it and then that lady also turns out to be suffering from something, then I say: ‘You could consider doing such a test, it could yield you some information that could help you.’, so yeah I’m quite enthusiastic about sharing. If I hear people talking about certain problems, I say: ‘Such a test could help you if you would want that.’.’ (P5, M54, HL* + *, NUM-, l.440-442)*	Q116
	*‘[Interviewer: ‘So you told me you shared your results and experiences with others’. Are you aware of them also potentially doing such a test themselves?’] Well, three of them at least. They all did such a test. [Interviewer: ‘Based on your recommendations?’] Yes.’ (P1, F59, HL* + *, NUM-, l.409-418)*	Q124
	*‘Yeah, and the reason why I also had my child tested is purely rational, you can do a lot with prevention, so I thought it would be better to know, regardless of what might potentially come out of it. Because then you can prepare yourself, or catch something early… I’ve also had them tested via one DTC-GT company, and I’m waiting for a sale from another company and then I’ll also have their DNA tested there, because they’re testing for very different markers.’ (P12, F42, HL* + *, NUM* + *, l.88 and l448)*	Q125
Satisfaction with DTC-GT consumer journey	*‘Well, definitely a 9… I’m fully satisfied. Due to this test I’ve gotten what I wanted.’ (P1, F59, HL* + *, NUM-, l.964-966)*	Q129
Suggestions by consumers to enhance the DTC-GT consumer journey	*‘Uh, yeah, I basically had to gather those bits of information myself, stuff like ‘What am I supposed to pay attention to?’. I think I got that from some article like: ‘What’s the points, quality requirements that I have to pay attention to when selecting a seller?, that was in an American scientific article or something. It would be handy if there was a website for that like: ‘If you’re considering undergoing DTC-GT, pay attention to this and that and that.’, and what also would’ve been handy was a list with what certain terms mean… It doesn’t matter who makes it [the website], as long as it’s made… It has to be good, reliable, maybe the Dutch Institute for Public Health and the Environment… Hmmm, and isn’t there an association of Dutch clinical geneticists {VKGN]? I think that would fit well.’ (P6, F61, HL* + *, NUM* + *, l.920-932)*	Q132
	*‘Uh, well, I found that part about the enzymes, look everyone knows what colon cancer is, those were clear-cut definitions. But with the enzyme part, you get ‘QZM and I-don’t-know-what’ enzymes and them I’m thinking: ‘I don’t know what that means.’, and then you can open it and read a bit more about that the enzyme plays a role in digestion or whatever, but that was a bit of ‘abracadabra’ sometimes, making me think: ‘Whatever.’… I think that was slightly hard to understand.’ (P8, F40, HL* + *, NUM-, l.214-218)*	Q135
	*‘Yeah, something from the seller like ‘…And always discuss these results with an expert, with a specialist.’… ‘If you still have questions or if something bothers you or something made you scared, please contact…’ and then I don’t know to what degree you can expect that from a seller but you know, I’m not sure with whom you should [make contact], whether that’s your GP, or the seller, or a specialist.’ (P5, M54, HL* + *, NUM-, l.582-588)*	Q136

^a^Legend: (participant, gender and age, health literacy high/low, numeracy high/low, lines from respective transcript from which quote was taken). Example Q1: (P3, F42, HL + , NUM-, l.80): quote by participant 3, a female aged 42 with high health literacy and low numeracy, taken from transcript line 80. Example Q53: (P15, M41, HL + , NUM + , l.32-34): quote by participant 15, a male aged 41 with high health literacy and high numeracy, taken from transcript lines 32-34.

### Pre-test

#### Varying initial contact sources and opinions regarding DTC-GT

Participants mentioned several sources through which they initially came across DTC-GT. Recurringly mentioned sources included prior consumers recommending the tests to them, exposure via regular media (e.g., TV and radio) as well as social media (e.g., Facebook), and alternative care HCPs (AC-HCPs: practitioners providing wellness- and health services outside of conventional care systems. Examples of AC-HCPs include as orthomolecular therapists, lifestyle coaches, psychosocial therapists and psychoneuroimmunologists) recommending the tests (Q1–Q4). Other, less-frequently mentioned routes included popular literature (e.g., magazines), Internet searches, targeted ad e-mails by DTC-GT companies, and (online) communities for e.g., donor children.

In general, initial opinions upon exposure to the concept of DTC-GT were described as rather positive, demonstrated by participants using terms such as ‘eye-opening’, ‘interesting’ and ‘exciting’ (Q5–Q6). A minority of participants indicated to have had more negative or ambivalent initial opinions, illustrated by labels such as ‘sceptic’ and ‘reserved’ (Q7–Q8).

#### Pre-test information acquisition

Topics for which participants actively sought information in the pre-test phase mainly pertained to test-related factors, such as logistics of the testing process, tested traits, possible actions that can be undertaken with test results, and test reliability. A minority of participants said to have obtained data-related and privacy-related information, for example about the option of removal of DNA data from sellers’ platforms. Sources most frequently mentioned for pre-test information acquisition were (websites of) DTC-GT sellers and testimonials of previous consumers either via word-of-mouth or review platforms. Other, less-frequently mentioned sources included AC-HCPs, consumer organizations, DTC-GT comparator websites, and TV documentaries (Q9–Q13).

#### Pre-test expectations: hopes, actions and consequences

When asked to recall their pre-test expectations, participants stressed that through DTC-GT, they mainly hoped to gain insight into their personal health traits, such as disease risk and drug sensitivity. They typically envisioned potentially acting on results by initiating lifestyle interventions such as changing diets, exercise regimen, or initiating supplementation. Other potential foreseen actions included warning family members of high disease risks and visiting HCPs (either AC-HCPs or conventional care HCPs (CC-HCPs: healthcare professionals providing health services within the regular healthcare system. Examples of CC-HCPs include GPs, clinical geneticists, oncologists and other types of registered and certified doctors)) for check-ups regarding disease risks and altered drug sensitivities reported by the DTC-GT. Another foreseen outcome was increased awareness of symptoms related to diseases for which participants were at high risk (Q14–Q19).

Most participants indicated that they wanted to obtain these insights mainly out of curiosity and general interest rather than perceived direct medical need (Q20–21). However, some also stressed they had wanted explanations for health issues that until then went unheard, unexplained, and/or inadequately responded to within the regular healthcare system. They emphasized a hope to obtain recognition and treatment (Q22–Q24).

Some participants said they had been specifically looking for insights into their carrier status for genetic diseases, which would be relevant in the context of reproductive decision making (Q25–Q27). Additionally, some participants noted they were primarily hoping to gain insight into their ancestry and heritage in addition to health-related insights, with two participants describing the health-related insights as ‘a byproduct’ (Q28–Q29).

In addition to these positive expectations, several participants noted they had been aware of the possibility of receiving (unexpected) indications of high disease risks, and that this could also result in fear and psychosocial impact. Furthermore, participants mentioned worries related to data safety and privacy, particularly concerning data leaks and (unwanted) usage and/or selling of data to/by third parties such as law enforcement or other stakeholders, as well as potential negative consequences for family members. Worries related to potential difficulties in obtaining insurance and/or mortgages due to DTC-GT results were also expressed (Q7, Q30–Q35).

#### Arguments driving decision-making

A variety of reasons driving participants’ decision-making processes were mentioned. Most arguments in support of undergoing DTC-GT were related to perceived benefits for participants themselves, but some reasons were more directly related to the test itself, such as a cheap price or temporary sale, or related to the positive opinions of others, such as acquaintances, relatives and/or HCPs, regarding participants considering undergoing DTC-GT. The opinions of HCPs were also said to impact decision-making more heavily. Participants also mentioned several arguments opposing undergoing DTC-GT. These considerations were often related to perceived harms or risks, such as data-related and finance-related worries. Mentioned examples included data leaks and selling of data to third parties, as well as difficulties regarding obtaining a mortgage or insurance in case DTC-GT reveals high disease risks. Other reasons included tests being perceived as expensive, and negative attitudes of acquaintances, relatives and/or HCPs towards participants considering undergoing DTC-GT. Several participants reported not having considered any potential negative consequences of undergoing DTC-GT during their decision-making process. Supporting arguments and opposing arguments mentioned by participants are shown in Table 3, together with supporting quotes illustrated in Supplementary Materials 3.

**Table 3:** Arguments mentioned by participants that impacted their decision-making process about undergoing health-related DTC-GT.

Arguments supporting undergoing health-related DTC-GT	Supporting quote(s)
Gaining insight into personal health-related factors to potentially act on, or at least be aware of increased risks	Q14–Q19
Satisfying curiosity and general interest in results without explicit desire for explaining health complaints that are currently unsatisfactorily resolved	Q20–Q21
Obtaining explanation/recognition/treatment for health complaints that were unsatisfactorily resolved within public healthcare system	Q22–Q24
Price-related factors (e.g. test was perceived as ‘cheap’, participants were persuaded by discounts or limited free trial periods, obtaining health-related insights in addition to heritage-related insights was relatively cheap)	Q36–Q40
Positive experiences of others with DTC-GT, either obtained directly via word-of-mouth or via other sources (e.g. Trustpilot)	Q41–Q44
Being able to share the experience of undergoing DTC-GT with someone else, or persuading them to also undergo it	Q45–Q46
Convenience and accessibility of testing	Q36, Q47
Positive attitudes of others (acquaintances, relatives, HCPs)	Q3, Q61–Q64
Wanting to know what it is like to undergo health-related DTC-GT before starting to advise/sell it to others	Q48
**Arguments opposing undergoing health-related DTC-GT**	
None considered	Q49–Q51
Data-related worries (e.g., data leaks, selling/usage of data to/by third parties)	Q7, Q52–55
Price-related considerations (e.g., test was perceived as ‘expensive’, participants stating they explicitly waited to buy a test until there was a sale going on)	Q39, Q56–58
Potential for (unexpected) high-risk results	Q30–Q31, Q59
Potential impact of undergoing DTC-GT for family members	Q33
Finance-related worries (e.g. high disease risk shown by DTC-GT could make obtaining a mortgage or insurance more difficult)	Q34–Q35, Q60
Negative attitudes of others (acquaintances, relatives, HCPs)	Q61–Q65

#### Consumers’ pathways and decision timelines

Participants mentioned seven different sellers from which they ultimately decided to buy DTC-GTs, six of which offered single nucleotide polymorphism (SNP) based testing, and one offered whole genome sequencing (WGS) based testing. Moreover, four participants decided to utilize a total of six different health-related third-party interpretation (TPI) services. Finally, three participants decided to make use of a combination of multiple health-related DTC-GTs and/or TPI services.

Participants described three main pathways through which they decided to obtain their health-related insights via DTC-GT. The first pathway was directly ordering tests from health-related DTC-GT sellers. The second was obtaining (additional) health-related insights either via TPI services offered by the original seller in the form of ‘health modules’, or via uploading their raw data to TPI services offered by other sellers or independent TPI service providers. The third was utilizing multiple unique DTC-GTs via different sellers. One participant said to have ultimately combined two routes, ordering both multiple DTC-GTs from different sellers, as well as uploading raw data of these tests to a health-related TPI service.

There was notable variation in the time intervals described by participants between first contact with the concept of DTC-GT and making the final decision to undergo health-related DTC-GT, ranging from hours and days, to months and years (Q66–Q67).

### Post-test

#### Post-test evaluation of test findings and insights

After physically doing the health-related DTC-GT and/or utilizing a health-related TPI service, participants generally reported looking at all their results immediately after they became available. Several participants expressed that their initial result-viewing behavior was significantly influenced by color coding and relative risk prioritization used in the information presentation (Q68–Q70).

Participants reported obtaining insights into a wide variety of health-related aspects via DTC-GT, including drug sensitivity, personalized genetic disease risk estimates, and other personal characteristics such as athleticism and food (in)tolerances. Multiple participants also reported receiving personalized lifestyle recommendations to act upon their health-related results (Q71–Q72).

Viewpoints regarding the understandability of the results differed markedly. Consumers of independent TPI services in particular reported initial difficulties in understanding their results (Q73–Q75). Differences in perceived understandability were also mentioned by different consumers that ordered the same test (Q76–Q77).

Differing perceptions regarding the reliability of their test results were mentioned by participants. Most participants said to have trusted the results because they recognized their own personal perceived health status and -risks in their results, had good faith in health-related DTC-GT providers, and/or had confidence in the HCPs that recommended DTC-GT to them (Q78–Q83). Some others expressed significant doubts about the reliability of their results (Q84–Q87). Several participants mentioned that their confidence in the reliability of the results diminished over time (Q86–Q87).

#### Post-test information acquisition

The degree of personal post-test information acquisition varied per participant. Some said to have read all post-test information provided by the seller, and additionally to have consulted academic experts in the field of human genetics and utilized online academic search tools. Others did not report any in-depth post-test information acquisition (Q88–Q89).

In addition to personal post-test information acquisition, several participants who bought a DTC-GT based on recommendation by an AC-HCP indicated that they had consulted these HCPs again after the test. These consumer journeys could all be linked to the same DTC-GT seller. Post-test services provided by these AC-HCPs ranged from providing support regarding interpretation of the results and general information provision, to giving personal (lifestyle) advice such as dietary changes and supplementation initiation and/or unlocking non-consumer accessible additional results (Q90–Q92). Some participants appeared to be under the impression that these AC-HCPs were actually CC-HCPs (Q92–Q93). Several participants stated that the AC-HCPs provided discount coupons for the supplements they advised to clients based on DTC-GT results (Q94).

Multiple participants reported sharing their results with CC-HCPs, for example to obtain personalized drug prescriptions, find diagnoses for unexplained disease, and validate or refute high disease risks and explore further implications (Q95–Q97). Some CC-HCPs were described as taking the results seriously and initiating referrals and follow-up testing, whereas other CC-HCPs were described as initially being dismissive and skeptical (Q97–Q100).

Participants who reported not having shared their results with CC-HCPs mentioned not doing so due to reasons related to not having noteworthy or worrisome high-risk results and not wanting to overload the regular healthcare system (Q101-Q102).

#### Health-related actions and experienced impacts

Changing diet and initiating supplementation were mentioned by several participants as implemented beneficial lifestyle changes based on DTC-GT results. These lifestyle changes were sometimes stimulated by post-test consultation of AC-HCPs (Q91, Q103). Other participants stated not having implemented lifestyle changes, mostly because they thought to already adhere to the recommended lifestyle. Others expressed seeing no additional benefit of implementing recommended lifestyle changes, which were often described as ‘generic’, in relation to personal disease risk (Q104–107).

Some participants reported to have utilized DTC-GT results in medical decision-making without consultation or approval by a CC-HCP. These actions included stopping taking medication that was earlier prescribed by CC-HCPs, and not getting vaccinated against COVID-19 (Q108–Q109).

Obtaining personalized medicine dosages (Q98), potential clinical confirmation of previously unexplained disease (Q97), reassurance (Q110–Q111), and general health improvements (Q91, Q103) were mentioned as positive experienced impacts of undergoing health-related DTC-GT and acting upon the results. Interestingly, two participants with self-reported personal and/or family history of a disease interpreted their negative SNP-array based results as implying that they were indeed not at increased familial risk of said disease, which they described as ‘relieving’ (Q110–Q111).

Negative experienced impacts were also brought up. These impacts mainly revolved around psychosocial impact, worry, and anxiety in response to results indicating high disease risks (Q112–Q114). One participant said to have pursued clinical-grade genetic testing to confirm these high disease risks, and that this testing revealed that the DTC-GT yielded false-positive results (Q114).

#### Sharing experiences and recommending testing to others

The degree to which experiences regarding health-related DTC-GT were shared outside of sharing with HCPs differed among participants. Some participants reported sharing their results and experiences with only close friends and family (Q115), others said they shared their experiences with anyone willing to hear (Q116).

Expressed adamancy of recommendation to others ranged from very strongly (Q116–Q117), to significantly more cautious (Q118–Q119). TPI service users said to be hesitant to recommend health-related DTC-GT via TPI services because of the difficulty of understanding results (Q120–122). One participant indicated having considered potentially buying DTC-GTs for employees as a Christmas gift (Q123). Multiple participants reported that others to whom they had recommended health-related DTC-GT, had indeed undergone it (Q124). The largest cascade of individuals that underwent DTC-GT following recommendations by a single interviewee included at least five other individuals.

Two participants indicated that they either intended to have their children, who were minors at the time of the interview, undergo DTC-GT, or had already done so (Q125–Q126). Additionally, one participant indicated that they had unlocked health-related insights for others without their knowledge and approval (Q127), and that they had not fully disclosed these results to these persons (Q128).

#### Satisfaction with DTC-GT consumer journey

Overall, high satisfaction with DTC-GT consumer journeys was reported, with all but two participants rating their experience a 6 or higher on a scale of 1–10. They indicated that they ultimately got out of it what they expected and/or wanted (Q129). Likewise, most participants indicated they would definitely do the test again if they could re-do their consumer journey.

Participants generally indicated they would not have changed anything about their undertaken consumer journey if they could have done so. However, some participants indicated that, in hindsight, they would have acquired more pre-test information before deciding to undergo health-related DTC-GT, for example by investigating differences between tests offered by various sellers (Q130).

#### Suggestions by consumers to enhance the DTC-GT consumer journey

When asked to look back on their consumer journey and pinpoint potential room for improvement, three recurring suggestions were made.

First, participants recommended augmenting of pre-test information provision. Proposed options included incorporating disclaimers of potential positive and negative consequences of undergoing health-related DTC-GT on sellers’ websites (Q131), facilitating information provision via sources perceived as reliable and independent, such as the Dutch National Institute for Public Health and the Environment, or the Dutch Clinical Geneticists’ Association (Q132), and having DTC-GT sellers install a customer service helpdesk that potential buyers can contact in case of questions (Q133).

The second suggestion concerned improving the test result communication. Participants expressed a desire for their results to be accessible in Dutch rather than English-only (Q134), and some would have liked more in-depth explanation of results and how to interpret those (Q135).

The final suggestion revolved around implementation of post-test support systems. Participants advocated for active calls to action by DTC-GT sellers to seek help if the results warranted so, for example in case of high disease risks. Participants differed in where they thought this help should and could be sought, ranging from the seller (Q136) to the regular healthcare system (Q137).

## Discussion

This study aimed to expand the knowledge regarding the perspectives, decision-making and experiences of non-US health-related DTC-GT consumers through studying consumer journeys of Dutch consumers. Overall, satisfaction regarding DTC-GT consumer journeys was substantial, in accordance with previous qualitative research [22–24]. A diversity of sources were implicated in participants’ initial contact with DTC-GT, as well as in their pre-test information acquisition. The pre-test expectations expressed by participants demonstrated considerable presumed clinical utility of DTC-GT. Multiple participants stated that feeling unheard or being inadequately responded to within the regular healthcare system supported their decision to undergo health-related DTC-GT, which has previously been reported in qualitative research from North-America and Australia [5, 25, 26], but appears to be a novel finding for a mainland European population. A modifying effect of price on decision-making was mentioned by a number of participants, and several participants reported not considering potential negative consequences of DTC-GT prior to DTC-GT usage. Several potentially adverse consequences of undergoing DTC-GT were identified that could affect individual consumers, the regular healthcare system, and society as a whole. These adverse consequences included (potentially unwarranted) distress and self-initiated medical decision-making based on DTC-GT results, as well as increased pressure on regular healthcare system resources, which have also been reported by previous case reports and qualitative studies [5, 22, 27, 28]. Finally, participants had several suggestions to enhance the DTC-GT consumer journey concerning pre-test information provision, result communication and post-test support systems. From these findings, we derived three considerations that could contribute to stimulating responsible use of DTC-GT while aligning with participants’ needs and preferences, namely improving pre-test information provision, implementing adequate post-test support systems for consumers, and development, implementation and enforcement of cross-border regulation and legislation. Here, these considerations will be presented and discussed.

### Consideration 1: Independent, reliable information provision via easily-accessible sources

Health-related DTC-GT sellers, (social) media, (testimonials of) previous consumers, and recommendation by AC-HCPs were identified as major initial contact and information sources regarding DTC-GT. The objectivity of information provided via these sources is likely doubtful as information provided by either sellers or (social) media has repeatedly been shown to be unbalanced and/or incomplete, inadequately empowering potential consumers for truly informed decision-making [29–34]. Moreover, consumers tend to be positively biased when reviewing a service they previously chose to purchase [35], providing biased testimonial-based information to potential future consumers. AC-HCPs, another important information source, can have a conflict of interest e.g. if they themselves receive a commission for selling the test or selling supplements based on test results.

Furthermore, our results indicate that throughout the consumer journey, there seem to be profound beliefs regarding the clinical utility of DTC-GTs among consumers, in-line with previous qualitative studies [5, 24]. For some participants, incorrect interpretations of DTC-GT results and/or their clinical utility led to (potentially unwarranted) medical decision-making, reassurance and/or other psychosocial impact. Previous qualitative and quantitative research has documented similar findings [5, 22, 28, 36, 37]. Although clinically-offered DTC-GTs such as pharmacogenetic tests [38] can be seen as an exception, the majority of commercial health-related DTC-GTs have not demonstrated clinical utility, with some sellers themselves even stating that their tests should only be used for informative purposes. As such, it is paramount that (potential) consumers are made aware of the limited clinical utility of commercial DTC-GTs.

Multiple participants also indicated not having considered any potential negative consequences of undergoing DTC-GT during the pre-test phase. This could be due to information acquisition via aforementioned biased sources, where potential consumers do not receive transparent information on potential risks and limitations of health-related DTC-GT.

Combined, these insights point towards considerable gaps in current pre-test information provision, in particular concerning the risks, limitations and current lack of proven clinical utility of commercial DTC-GTs. Since enhancement of pre-test information provision was also recommended by participants themselves, as well as previously by Dutch citizens that had not (yet) undergone DTC-GT [39], this approach would seem to conform well with the population’s preferences and needs, suggesting a degree of societal alignment [11]. To ensure optimal societal alignment and efficacy however, interventions in accordance with this approach must be designed utilizing dialogue-focused methods, such as the mental models approach, rather than deficit models [11, 40, 41]. One option could be implementation of co-created (social) media campaigns concerning DTC-GT, employing for example scientists from governmental organizations or CC-HCPs, since participants suggested them as suitable stakeholders for independent information provision. Indeed, (social) media campaigns in other public health related contexts showed notable effects on health-related decision-making and behavior [42, 43]. Through such an approach, potential DTC-GT consumers can be reached with unbiased information that is adjusted to common lay perceptions and beliefs via easily-accessible sources that (potential) consumers already utilize for pre-test information acquisition.

### Consideration 2: Education of CC-HCPs

After receiving their test results, initial result interpretation by consumers seemed to be largely influenced by the color coding and relative risk prioritization that is often used by DTC-GT sellers as a risk categorization approach. This form of presenting and grouping results is likely complex for consumers, especially those with low health literacy and/or numeracy [44]. Indeed, various participants indicated initial troubles with understanding their results. These troubles might prompt consumers to utilize additional resources to help them interpret the results. However, it is known that DTC-GT sellers themselves often do not extensively provide these resources, with only a third of companies offering post-test counseling, sometimes with additional fees [30].

Consequently, HCPs could be a source that consumers might turn to for help. Indeed, a wide variety of CC-HCPs are known to have been consulted about questions regarding DTC-GT by consumers, including genetic specialists such as clinical geneticists and genetic counselors, but also non-geneticists such as GPs, oncologists and nurse practitioners [6]. However, suboptimal knowledge and confidence levels regarding health-related DTC-GT and DTC-GT related counseling have been documented among various types of CC-HCPs [45]. Additionally, consumers have previously reported dissatisfaction with counseling by and attitudes of CC-HCPs in the context of DTC-GT [5]. These findings suggest CC-HCPs might currently, in various ways, be inadequately equipped to satisfactorily respond to consumers’ questions and concerns. This could also explain the remarkable proportion of our study participants that underwent counseling for their DTC-GT results with AC-HCPs rather than CC-HCPs. This process could exacerbate the risk of misinterpretation and its potential consequences such as (unwarranted) psychosocial impact, adverse medical decision-making and overloading of regular healthcare systems.

To mitigate these risks, education of CC-HCPs seems warranted, as also previously suggested [45]. This education should not only focus on improving the efficacy of CC-HCPs regarding interpretation of DTC-GT results, but also on counseling worried consumers, to ensure that the needs of these consumers are properly addressed. In addition, development of guidelines for counseling of patients presenting with DTC-GT results could be beneficial since CC-HCPs likely rarely encounter these patients given the relatively small proportion of DTC-GT consumers in several countries, including the Netherlands [6, 15]. Again, although participants appeared to support this suggestion through recommending implementation of adequate post-test support systems, the contents of these educational modules should be developed utilizing dialogue-based models such as mental models approaches to ensure the needs of consumers seeking help are properly met, striving for efficacy and societal alignment [11, 40, 41].

### Consideration 3: Development, implementation and enforcement of cross-border regulation and legislation

Realizing participants’ suggestions concerning improved test result communication and implementation of post-test support systems could be achieved by means of developing and implementing cross-border regulation and legislation concerning DTC-GT services, and subsequent enforcement of implemented policies. For example, regulation and legislation demanding a certain quality of the tests themselves, but also mandating sellers to provide pre-test and post-test counseling could be beneficial. This would be advantageous for both consumers as well as the regular healthcare system through preventing unwarranted medical and psychosocial impacts, as well as unnecessary usage of healthcare resources.

However, there is uncertainty regarding roles and responsibilities of individual stakeholders involved in making, implementing, and enforcing policies pertaining to DTC-GT services. This is illustrated by the fragmented regulatory and legislative frameworks currently employed by different countries: while some regulations and legislations pertaining partially to DTC-GT exist, such as the ‘In-Vitro Diagnostics Regulation’ (IVDR), most countries are currently lacking in enforceable policy specific to DTC-GT services [2, 46, 47]. The international and dynamic character of the DTC-GT market [48] further hampers enforcement of policies.

Thus, there are several important factors that have to be taken into account. Firstly, roles and responsibilities of individual stakeholders involved in making, implementing, and enforcing policies related to DTC-GT services need to be clarified, as well as the elements of the DTC-GT services that are to be targeted by policies to ensure the minimum quality and contents of these services. Recently developed evaluation frameworks for DTC-GT could aid in those tasks [2]. Secondly, it is paramount that policies are not only implemented nationally and regionally, but rather at a worldwide level. This would enable universal enforcement, ensuring similar quality standards for DTC-GT services worldwide, eliminating the current fragmentation of the policy landscape [46]. Through these approaches, the minimum quality and contents of DTC-GT services can be properly ensured and enforced, corresponding with participants’ needs for improved test result communications and implementation of adequate post-test support systems.

### Strengths and limitations

This study contributes to filling an important knowledge gap often referenced in literature, by studying the perspectives of health-related DTC-GT consumers outside of the US [3, 5, 6] with a sufficient sample size to provide robust qualitative insights [17].

The study may be limited by the use of convenience sampling, with participants who were willing and eager to participate. As such, it is possible that the participants are either considerably more positive or negative than the average consumer.

Another potential limitation is that the study population consisted almost exclusively of participants with relatively high health literacy. Although the proportion of DTC-GT consumers with limited health literacy is unknown, it is known that approximately a third of Dutch citizens have limited health literacy [49]. As such, it is possible that participants with high health literacy were over-represented in our study population compared to the demographics of the total Dutch DTC-GT consumer population. Given the importance of health literacy in medical- and health-related decision-making [50], it is important that the views and experiences of DTC-GT consumers with lower health literacy are also studied.

## Conclusion

To safeguard and stimulate responsible use of health-related DTC-GT, improving pre-test information provision, implementing adequate post-test support systems for consumers through education of CC-HCPs, and development, implementation and enforcement of cross-border regulation and legislation should be considered. Although further quantitative confirmation of our findings is warranted, we advocate for in-depth exploration of these considerations with all involved stakeholders, such as the general public, DTC-GT consumers, HCPs, policymakers and regulators, as well as the industry itself. Through this approach, deliverables stimulating responsible use of DTC-GT as well as corresponding implementation- and dissemination strategies for these deliverables can be developed through co-creative efforts, ensuring they are in-line with stakeholders’ needs and preferences. Ultimately, these deliverables are thus envisioned to aid in empowering consumers for responsible use of DTC-GT.

## Supplementary information

Supplementary Material 1: Topic Guide

Supplementary Material 2: Codebook

Supplementary Material 3: Supporting Quotes

## Data Availability

To protect the anonymity of participants, interview transcripts will not be shared. Information about the materials utilized in the interviews can be found in the **Supplementary Materials**, such as the topic guide (Supplementary Materials 1), the codebook (Supplementary Materials 2) and the extensive quotes table (Supplementary Materials 3). Requests for and questions about the data utilized for this manuscript can be addressed to the corresponding author.
