# Supplementary material for: Navigating direct-to-consumer genetic testing: experiences, decisions and perspectives of Dutch users
Source: Eur J Hum Genet. 2026 Feb 4;34(4):480–90. doi: 10.1038/s41431-026-02022-z (PMC13046840; doi:10.1038/s41431-026-02022-z)
Supplement: Supplementary file 1 — Supplementary Material 1: Topic Guide [file 41431_2026_2022_MOESM1_ESM.docx]

**Navigating direct-to-consumer genetic testing: experiences, decisions and perspectives of Dutch users**

**European Journal of Human Genetics**

**Author information**

***Danny Bruins^a^, Esther A.M. Bührman^a^, Martina C. Cornel^a^, Margreet G.E.M. Ausems^c^, Marc H.W. van Mil^d,e^, Olga C. Damman^f^, Tessel Rigter^a,b^***

^a^Section Community Genetics, Department of Human Genetics, Amsterdam Public Health Research Institute, Personalized Medicine, Amsterdam UMC, Vrije Universiteit Amsterdam, 1105 AZ Amsterdam, The Netherlands.

^b^Center for Health Protection, National Institute for Public Health and the Environment, 3721 MA Bilthoven, The Netherlands.

^c^Department of Genetics, Division Laboratories, Pharmacy and Biomedical Genetics, University Medical Center Utrecht, 3584 CX Utrecht, The Netherlands.

^d^Center of Education and Training, University Medical Center Utrecht, 3584 CX Utrecht, The Netherlands.

^e^Center for Molecular Medicine, University Medical Center Utrecht, 3584 CX Utrecht, The Netherlands.

^f^Department of Public and Occupational Health, Amsterdam Public Health Research Institute, Quality of Care, Amsterdam UMC, Vrije Universiteit Amsterdam, 1105 AZ Amsterdam, the Netherlands.

**Corresponding Author:** Danny Bruins, d.bruins@amsterdamumc.nl

**Funding:** This study was conducted as part of the ERUDIGIT project, funded by Netherlands Organisation for Health Research and Development, grant number 05550402110010.

**Supplementary Materials 1: Topic Guide**

The English translation of the Dutch topic guide that was utilized during the interviews can be found underneath.

*You have done a DNA home test for health. We will start with some questions about this test, and the period before you took the test. So, I’d like to ask you to think back to that period.*

**I. Do you remember which specific DNA health home test you took?**

- Do you remember the name of the provider? Or what the brand was?
- Did you purchase the test yourself, or did you receive it as a gift?
- What kind of test was it, and what was it for?

**II. How long ago did you take this test?**

**III. How did you first come into contact with DNA home tests for health?**

- What did you think about it at the time?
- Has your opinion changed over time? If so, why?

**IV. Can you tell me why you considered taking this test? Did you have any specific reasons for doing it? If yes, what were these reasons?**

- Were there any other reasons?
- Were there also possible reasons not to take the test, or reasons you hesitated? What were they?
- How quickly did you decide to purchase the test (or do it if you received it)? Were you immediately convinced, or did you have doubts? What made you feel that way?

**V. What is the most important reason why you took the test, if you would say it in your own words now?**

- Can you explain why that was the most important reason?

**VI. What information was relevant/important to you when deciding to do a DNA home test for health? What did you base your decision on? Do you still remember that?**

- What topics did this information cover?
- Where did you get this information?
- Did you look for any extra information that didn’t come from the seller or test maker? What, and where?
- Was there any information you would have liked to have had before deciding to take the DNA home test, but couldn’t find? If yes, what?
- Were you satisfied with how you could find the information and how it was presented, or do you think it could have been better? What made you feel satisfied or not?

**VII. Did you discuss your intention to take the test with anyone? If yes, who?**

- What did they think? What did you discuss?
- Did those conversations play an important role in your decision to take a DNA home test for health?

**VIII. What expectations did you have about the test beforehand?**

- How did you think the results would look?
- What did you expect the test to give you? What were you planning to do with the test result in advance?
- What positive and/or negative consequences did you foresee from taking such a DNA home test?

**IX. What was your predominant feeling when you had just done the DNA home test and sent it to the provider?**

*We now move on to the period after you did the DNA home test and received the results. I would like you to think back to that period now.*

**I. How did you feel or what did you think when you heard that the results were ready?**

**II. What kind of results did you get back?**

- How did you receive the results?
- Can you walk me through how the results looked? You heard that the results were ready, you opened them, and then what? What did you see? What stood out to you?
  - How did you experience this?
  - Did you immediately look at all the results, or did you let some of them sit for a while?
  - Did you look at all the scores, or just specific ones?
- Did you find the results easy or difficult to understand? Can you explain?
  - What did you find easy or difficult about it?
  - Were there things you would have preferred to see done differently to make the results easier to understand? If so, what?
  - How reliable did the results, the reasoning behind them, and other information you received with the results seem? Why?
- Did the results look different from what you had expected beforehand? If so, what was the difference?

**III. What emotions did the results evoke in you? How did you feel when you received the results?**

- Did your feelings about the results change over time? Did they become stronger, weaker, or change? Are you now more or less concerned/satisfied than you were at first?

**IV. What actions did you take based on the test results? Did you do anything with them?**

- Did you talk to anyone or ask someone for advice about the results? Provider, doctor, family, partner?
- Did you seek additional information based on the results? About what? Where did you find this info?
  - Did you contact the seller about the results?
- To what extent did you change your lifestyle/behavior based on the results? If yes: what & how?
- Did you do what you had expected to do with the test result beforehand?
  - If not: why not?
- Was there a difference between what you expected the test to give you versus what it actually gave you?
  - If yes: what was the difference?
- To what extent did a healthcare provider play a role in your actions or inaction? This could have been before or after the test.
  - If yes: which healthcare provider, and what actions?

**V. What positive and/or negative consequences have you experienced from taking the test?**

- Did you face any of the possible consequences you expected before taking the test after completing it?
- Were there any consequences you didn’t expect before the test, but faced after?

**VI. Were there any aspects we haven’t covered yet where your experience with the DNA home test for health didn’t match your expectations beforehand?**

**VII. Looking back at your overall experience with a DNA home test for health, how satisfied are you with it on a scale of 1-10?**

- Why that score?
- What are you satisfied or not satisfied with? What could have been better?
- Do you regret taking the DNA home test?
- Would you, based on your experience, do the DNA test again if you could go back in time?
  - Why/why not? Have you become more positive/negative about the test than before you took it?
  - Would you recommend it to someone else? Why/why not?
- If you were to do the test again, would you do anything differently (before or after)?

**VIII. Reflecting on your experience, did you miss any support/help/information when making the decision about whether or not to do a DNA home test for health? Would you have needed it?**

- What kind of extra help would you have liked to receive?
- How do you envision that support/help?
- Who do you think should provide this support/help?

*We have now reached the end of the interview!*

**I. Could you briefly summarize your opinion on DNA home tests and how you arrived at that opinion? Are there any points you think are important to mention?**

- Would you like to add anything to what you have said?
- How did you find participating in this interview?
- Do you have any questions at the moment?

*Thank you very much for your participation, I will now end the recording.*
