# Supplementary material for: Navigating direct-to-consumer genetic testing: experiences, decisions and perspectives of Dutch users
Source: Eur J Hum Genet. 2026 Feb 4;34(4):480–90. doi: 10.1038/s41431-026-02022-z (PMC13046840; doi:10.1038/s41431-026-02022-z)
Supplement: Supplementary file 2 — Supplementary Material 2: Codebook [file 41431_2026_2022_MOESM2_ESM.docx]

**Navigating direct-to-consumer genetic testing: experiences, decisions and perspectives of Dutch users**

**European Journal of Human Genetics**

**Author information**

***Danny Bruins^a^, Esther A.M. Bührman^a^, Martina C. Cornel^a^, Margreet G.E.M. Ausems^c^, Marc H.W. van Mil^d,e^, Olga C. Damman^f^, Tessel Rigter^a,b^***

^a^Section Community Genetics, Department of Human Genetics, Amsterdam Public Health Research Institute, Personalized Medicine, Amsterdam UMC, Vrije Universiteit Amsterdam, 1105 AZ Amsterdam, The Netherlands.
^b^Center for Health Protection, National Institute for Public Health and the Environment, 3721 MA Bilthoven, The Netherlands.
^c^Department of Genetics, Division Laboratories, Pharmacy and Biomedical Genetics, University Medical Center Utrecht, 3584 CX Utrecht, The Netherlands.
^d^Center of Education and Training, University Medical Center Utrecht, 3584 CX Utrecht, The Netherlands.
^e^Center for Molecular Medicine, University Medical Center Utrecht, 3584 CX Utrecht, The Netherlands.
^f^Department of Public and Occupational Health, Amsterdam Public Health Research Institute, Quality of Care, Amsterdam UMC, Vrije Universiteit Amsterdam, 1105 AZ Amsterdam, the Netherlands.

**Corresponding Author:** Danny Bruins, d.bruins@amsterdamumc.nl

**Funding:** This study was conducted as part of the ERUDIGIT project, funded by Netherlands Organisation for Health Research and Development, grant number 05550402110010.

Supplementary Materials 2: Codebook

The codebook with which data analysis was performed can be found underneath.

| Code System |  |
| --- | --- |
| DTC-GT |  |
| Brand & Type | Participant remarks brand and type of DTC-GT he/she has done and/or third party analyzer utilized |
| Price | Participant makes remarks concerning the price of the DTC-GT they have done |
| Time since undergoing DTC-GT | Participant remarks how long ago they have done a health-related DTC-GT |
| Option to unlock additional modules | Participant mentions that for the respective DTC-GT that they have done, additional modules can be unlocked |
| Using multiple DTC-GTs and/or TPIs | Participant mentions that they underwent multiple health-related DTC-GTs, or uploaded their raw DTC-GT data to third party analyzers (TPIs) |
| Pre-test |  |
| Initial contact with DTC-GT | Participant explains how they first came into contact with DTC-GT (e.g. via a person, website, ad, newspaper, etc.) |
| Initial opinion regarding DTC-GT | Participant explains what they initially thought of DTC-GT when they first came into contact with it |
| Information | Participant makes remarks regarding pre-test information |
| Obtained information | Participant makes remarks regarding information they OBTAINED pre-test. |
| Topics | Participant mentions topics for which they gathered information to support their decision making process (e.g. 'tested variants/traits', 'turnaround time', 'price of testing', 'quality of test', 'looked at multiple tests', 'privacy-related aspects', etc.) |
| Sources | Participant remarks informational sources they used for their decision making regarding undergoing health-related DTC-GT (e.g. Google, HCP, thuisarts.nl, reviews of prior consumers, etc.) |
| Persons | Participant makes remarks regarding persons through which they obtained pre-test information regarding DTC-GT (e.g. relatives, acquaintances, HCPs, etc.) |
| Media | Participant remarks which media they used to obtain pre-test information regarding DTC-GT |
| Regular media | Participant remarks that they used regular media to obtain pre-test information regarding DTC-GT (e.g. radio, TV, internet, newspaper, etc.) |
| Social media | Participant remarks that they utilized social media to obtain pre-test information about DTC-GT |
| Literature | Participant makes remarks regarding literature they used to obtain pre-test information regarding DTC-GT |
| Academic | Participant states that they obtained pre-test information regarding DTC-GT through academic literature (e.g. PubMed articles, books by academics, etc.) |
| Non-academic | Participant remarks that they utilized non-academic literature to obtain pre-test information regarding DTC-GT (e.g. certain books that can be described as non-academic). |
| Missed information (before DM in consumer journey) |  |
| Topics | Participant mentions topics for which they MISSED information to support their decision making process (e.g. 'tested variants/traits', 'turnaround time', 'price of testing', 'quality of test', 'looked at multiple tests', 'privacy-related aspects', etc.) |
| Sources | Participant remarks informational sources they MISSED before/during their decision making regarding undergoing health-related DTC-GT (e.g. Google, HCP, thuisarts.nl, reviews of prior consumers, etc.) |
| Persons | Participant makes remarks regarding persons through which they MISSED being able to obtain pre-test information regarding DTC-GT (e.g. relatives, acquaintances, HCPs, etc.) |
| Media | Participant remarks which media they MISSED being able to use to obtain pre-test information regarding DTC-GT |
| Regular media | Participant remarks that they MISSED being able to use regular media to obtain pre-test information regarding DTC-GT (e.g. radio, TV, internet, newspaper, etc.) |
| Social media | Participant remarks that they MISSED being able to utilize social media to obtain pre-test information about DTC-GT |
| Literature | Participant makes remarks regarding literature they MISSED to obtain pre-test information regarding DTC-GT |
| Academic | Participant states that they MISSED being able to obtain pre-test information regarding DTC-GT through academic literature (e.g. PubMed articles, books by academics, etc.) |
| Non-academic | Participant remarks that they MISSED being able to utilize non-academic literature to obtain pre-test information regarding DTC-GT (e.g. certain books that can be described as non-academic). |
| None | Participant states they missed no pre-test information looking back on their decision-making process |
| Satisfaction | Participant makes remarks about their satisfaction regarding pre-test information provision |
| Pre-Test Expectations | Participant mentions something about their pre-test expectations regarding testing |
| Test kit contents & logistics | Participant makes remarks about the physical contents of test kit and logistics of DTC-GT (e.g. how they had to send the test back to the seller, turnaround time of results, etc.) |
| Test Results & Recommendations | Participant makes remarks about their pre-test expectations regarding what the test entails, and what they will get back from the test (e.g. in terms of results, knowledge, or personalized recommendations, etc.) |
| Foreseen actions | Participant makes remarks concerning actions they foresaw to undertake using their DTC-GT results before doing the test |
| Foreseen impact | Participant remarks certain impacts they foresaw doing a DTC-GT could have for them (e.g. discovering high disease risk, insurability problems, receiving personalized medicine dosage recommendations) |
| Positive | Participant mentions positive pre-test foreseen impacts of undergoing (health-related) DTC-GT (e.g. 'receiving personalized medicine', 'getting access to certain budgets due to receiving a diagnosis', 'quenching curiosity towards familial disease risk') |
| Negative | Participant mentions negative pre-test foreseen impacts of undergoing (health-related) DTC-GT (e.g. 'unwanted impact on family', 'risk of getting unexpected high-risk results', 'impact on insurability', 'impact on getting a job', etc.) |
| Argumentation & Decision Making, Motivation |  |
| Reasons considered supporting undergoing DTC-GT | Participant mentions reasons for why they initially considered undergoing health-related DTC-GT (e.g. 'part of package with ancestry test', 'low additional price on top of price for ancestry test', 'getting sick', 'not trusting regular healthcare', 'not being taken seriously within regular healthcare', 'regular healthcare failed to explain my ailments', 'wanting personalized medicine', 'temporary discount', etc.) |
| General curiosity | Participant states 'general curiosity' as an argument that supported their undergoing of DTC-GT |
| Increased knowledge | Participant states 'increased knowledge' as reason supporting their undergoing of DTC-GT (e.g. 'Knowledge is power', etc.) |
| Insight in health-related factors | Participant states 'insight in health-related factors' as an argument that supported their undergoing of DTC-GT. (e.g. personalizing drug doses, being able to take preventive measures towards high-risk diseases, etc.) |
| Heritage-related questions | Participant states 'questions concerning heritage' as an argument that supported their undergoing of DTC-GT (e.g. 'I wondered if I was partially English', etc.) |
| Positive experience of others with DTC-GT | Participant states 'Positive experience of others' as an argument that supported their undergoing of DTC-GT (e.g. 'My relative/friend did a test and was enthusiastic, so I wanted to do it as well!', etc.) |
| Sharing experience with someone else | Participant states 'sharing experience with someone else' as an argument that supported their undergoing of DTC-GT (e.g. 'My spouse wanted to do a test, so I decided to do one as well to be able to compare results and gauge accuracy', etc.) |
| Needs not met by regular healthcare | Participant states 'needs not met by regular healthcare' as an argument that supported their undergoing of DTC-GT (e.g. 'doctor says there's nothing wrong, but I feel sick', 'They didn't want to treat me', 'They didn't want to personalize my drug dosage', etc.) |
| Price | Participant states 'price' as an argument that supported their undergoing of DTC-GT (e.g. 'There was a temporary discount', 'I thought the test was cheaper than going through regular healthcare to undergo genetic testing', etc.) |
| Wanting to know what you're advising others | People state that they underwent DTC-GT because they wanted to know what exactly the test entails before they're advising it to others (e.g. because participant is an alternative HCP). |
| Convenience/accessibility | Participant mentions convenience/accessibility-related statements as supporting their decision making: 'it's easy to do', 'you don't have to leave the house', 'you can do it from the couch', etc. |
| Reasons considered against undergoing DTC-GT | Participant mentions reasons they considered (or could foresee) against undergoing health-related DTC-GT (e.g. 'not wanting to know certain things', 'price', 'results could be unreliable', 'insurability problems' etc.) |
| Family-related issues | Participant states that they took family-related issues (e.g. impact of doing DTC-GT or certain discoveries that can result from doing DTC-GT) into account during decision-making. |
| Risk/fear of unexpected results | Participant states 'risk/fear of unexpected results' as an argument that argued against their undergoing of DTC-GT (e.g. 'I was worried about receiving unexpected high-risk results', etc.) |
| Data-related issues | Participant states 'data-related issues' as an argument that argued against their undergoing of DTC-GT (e.g. 'I was unsure where my DNA data would end up if I underwent DTC-GT, or what the company would do with my data', 'What would happen to my data in case the company went bankrupt', etc.) |
| Finance-related issues (insurance, mortgage) | Participant states 'finance-related issues' (e.g. mortgage, insurance-related) as an argument that argued against their undergoing of DTC-GT (e.g. 'Undergoing DTC-GT could mean problems with getting insurance if you get back a high-risk result', etc.) |
| Price | Participant states 'price' as an argument that argued against their undergoing of DTC-GT (e.g. 'The test was quite expensive', etc.) |
| None | Participant states that they did not consider any arguments against undergoing DTC-GT during their decision-making process |
| Key reason supporting decision making | Participants mention the key reason that made them decide to undergo health-related DTC-GT |
| Discussion with others | Participant remarks they had pre-test discussions/conversations with others about intention to test, and what was discussed then. |
| Time between first contact DTC-GT and deciding to do DTC-GT | Participant remarks how much time they spent between first coming into contact with DTC-GT, and actually doing the test |
| Post-test |  |
| Emotions | Participant describes emotions they felt somewhere after doing the DTC-GT |
| Emotions when sending in testing kit to lab | Participant makes remarks regarding the feelings they had when sending in their test kit to the lab (e.g. 'excited', 'impatient for receiving results', 'scared/afraid', etc.) |
| Emotions & change thereof over time | Participant makes remarks concerning the emotions they felt when they heard their DTC-GT results were available and emotions about their results overall, and how these emotions changed (or didn't) over time |
| Results | Participant makes remarks concerning the results they received from doing a DTC-GT |
| Raw data | Participant remarks that they also have access to raw data from their DTC-GT |
| Lifestyle recommendations | Participant makes remarks about if and which lifestyle recommendations the DTC-GT they did provides |
| Tested variants/traits | Participant makes remarks about which traits they received results for from their DTC-GT (e.g. pharmacogenomics, disease risk, athleticism, stress, heritage, etc.) |
| Locked results | Participant makes remarks that some DTC-GT results were initially locked, but could manually be unlocked by consumer |
| Disclaimers | Participant remarks that there were disclaimers by the company accompanying their results (e.g. 'If you get a positive result, this doesn't mean you'll actually get the disease', 'If you get a negative result, this doesn't mean you will not get the disease', 'The results of this test should not be used medically, always contact a GP prior to acting on these results', etc.) |
| Understandability | Participant makes remarks regarding how easy/hard they found their DTC-GT results to understand (e.g. 'I found it intuitive/easy', 'I would rather have seen it another way', 'I found it hard to understand', etc.) |
| Perceived reliability | Participant makes remarks concerning how reliable they perceive their results to be (e.g. mentioning that 'they're happy with sources/references that they get with their results', 'I think they're reliable', 'Multiple sources have provided me with this finding now, so I trust the DTC-GT result is true', etc.) |
| Perceived utility of results | Participants makes remarks indicating how they perceive the utility of health-related DTC-GT results (e.g. 'I'll only know how reliable these results are once I've already developed a disease', 'I feel like getting these results enables me to take more control of my life through medication/lifestyle/whatever', 'It's fun to know, but not very useful since you can't prevent developing certain traits such as Alzheimer's', etc.) |
| Updates by company | Participant remarks that DTC-GT seller occasionally provides updates regarding results (e.g. (small) alterations of disease risk). |
| Satisfaction regarding results | Participant makes remarks concerning their satisfaction about results they have received (e.g. 'I got more than I hoped for', 'I was satisfied with what I got', etc.) |
| Actual Experience vs. Pre-Test Expectations | Participant makes remarks comparing their experience against their pre-test expectations |
| Experience w/ & Actions after getting DTC-GT results | Participant makes remarks regarding how they (tried to) use(d) findings from their health-related DTC-GT |
| Using test kit & test process overall | Participant makes remarks concerning their experience with using the DTC-GT test kit and het overall testing process (e.g. 'easy to take sample', 'low turnaround time', 'easy to reach the company in case you have questions', etc.) |
| Gathering additional information | Participant states that they, gathered additional information about disease/traits/genetics, whatever, based on their DTC-GT results (e.g. via click-through links to PubMed in DTC-GT apps, searching on Internet/reading books on own initiative) |
| Initial result viewing behavior | Participant remarks how they initially started going through their health-related DTC-GT results (e.g. 'all at once', 'prioritizing ones I was initially interested in, looking at the others later', 'focusing on results that came up as high-risk/aberrant/not-normal', 'not very in-depth, glossed over it', etc.) |
| Result interpretation by consumers | Participant makes remarks indicating how they interpret (some of) their results |
| Making lifestyle changes | Participant makes remarks regarding whether they used their DTC-GT results to inspire lifestyle changes (e.g. becoming more active, taking (different) supplements, making changes in diet, etc.) |
| Doing additional tests | Participant states they had undergone additional (self)-tests due to their DTC-GT results |
| Sharing results with others (non-HCP) | Participant makes remarks about whom, where, what they shared their DTC-GT results with others that were not healthcare 'professionals'. |
| Recommending DTC-GT to others | Participant remarks (directly or indirectly) that they recommended undergoing DTC-GT to others |
| Using/sharing results in regular health care system | Participant makes remarks concerning how they (tried to) use/share results from their DTC-GT in regular healthcare system (e.g. at pharmacy, hospital, GP, etc.), and how positive/negative this experience was with regards to the (perceived) attitude of the HCP towards the consumer and their DTC-GT results |
| Positive experience (perc HCP attitude) | Participant remarks that HCPs in regular healthcare were positive regarding DTC-GT results (e.g. 'taking them seriously', 'initiation additional testing', 'changing drug dosage based on DTC-GT results', etc.) |
| Negative experience (perc HCP attitude) | Participant remarks that HCPs in regular healthcare were negative regarding DTC-GT results (e.g. 'not taking them seriously', 'being dismissive', 'not trusting results', etc.) |
| Neutral experience | Participant remarks sharing their DTC-GT results within regular healthcare, without remarking (directly or indirectly) whether this experience was positive or negative |
| Results not shared in regular healthcare system | Participant mentions they did not share their results within the regular healthcare system |
| Experienced effects/impact of doing health-related DTC-GT | Participant mentions experienced effects/impact of doing health-related DTC-GT, and whether these were positive, negative, or neutral |
| Positive | Participant mentions positive effects of undergoing health-related DTC-GT (e.g. receiving personalized medicine dosage, quenching curiosity, increased knowledge of self, re-assurance of low disease risks, awareness of high disease risks, etc.) |
| Negative | Participant mentions negative effects/impact of undergoing health-related DTC-GT (e.g. 'feeling scammed', 'unnecessary anxiety', 'exposure to unexpectedly increased risk', 'false reassurance', 'insurance problems', 'people think it's weird I did this, they don't trust it and ask why' etc.) |
| Neutral/None | Participant states they did not experience any effect/impact of undergoing health-related DTC-GT (e.g. 'It didn't change anything for me', 'It didn't impact my life in any meaningful way', 'I didn't do anything with it', etc.) |
| Things people would do differently if possible | Participant remarks whether they would do something differently in their consumer journey than they actually did if they could go back in time. |
| Test rating | Participant rates their experience with undergoing DTC-GT |
| Grade | Grade participant gives their overall experience with undergoing DTC-GT (e.g. 'an 8, I was satisfied', etc.) |
| Intention to test again if participant could go back in time | Participant remarks whether they would do DTC-GT again if they could go back in time or not |
| Roles of stakeholders in DTC-GT consumer's journey | Participant makes remarks concerning a healthcare 'professional' that helped them somewhere in their consumer journey (e.g. through consults regarding DTC-GT, providing information regarding DTC-GT, etc.). HCP can be either a 'professional' (e.g. orthomolecular therapists), but also a 'true HCP'. |
| Pre-test information provision | A stakeholder provided participant with information regarding DTC-GT pre-test |
| Pre-test discussion about participant wanting to do DTC-GT | Some stakeholder took part in a discussion/conversation with participant about participant wanting to undergo DTC-GT |
| Stimulating/nudging towards DTC-GT usage | Some stakeholder stimulated or nudged participant to undergo DTC-GT, e.g. through recommending DTC-GT to them either directly or indirectly (through e.g. information provision), sellers offering ‘temporary’ discounts, etc. |
| Dissuading from DTC-GT usage | Some stakeholder dissuaded participant to undergo DTC-GT, e.g. advising them to not undergo DTC-GT either directly or indirectly (through e.g. information provision by government), etc. |
| Test interpretation | A stakeholder helped with interpretation of participant's DTC-GT results |
| Giving recommendations based on test results | Some stakeholder gave recommendations based on test results (e.g. changing lifestyle, diet, supplements, certain treatment/test in hospital, etc.) |
| Unlocking non-consumer accessible additional results | Participant remarks that a certain stakeholder has to unlock certain results that consumers cannot unlock themselves. |
| Post-test information provision | Some stakeholder provided participant with post-test information in the context of DTC-GT, e.g. seller linking to relevant PubMed publications, relative recommending them to read certain literature/books/podcasts |
| Post-test consultation (unspecified) | Some stakeholder provided general post-test consultation to DTC-GT consumer (e.g. within regular healthcare) |
| Performing/initiating additional (self)tests | Some stakeholder performed/initiated additional tests based on DTC-GT results |
| Was recommended DTC-GT by participant | Some stakeholder was recommended to undergo DTC-GT by participant (e.g. advising them to undergo DTC-GT due to suffering from certain (unexplained) complaints for some time, etc. |
| Stakeholder involved in consumer's DTC-GT journey | Participant describes (a) stakeholder(s) involved in some point of their consumer journey |
| Acquaintance | Anyone that isn't a relative, HCP, or (employee of) DTC-GT seller |
| Relative (family) |  |
| HCP |  |
| Alternative healthcare HCP |  |
| Regular healthcare HCP |  |
| DTC-GT seller |  |
| Other | Anyone you wouldn't simply describe as 'acquaintance' because it feels too 'thin' for the roles they fulfilled in someone's consumer journey, but who isn't a HCP, seller, or relative. |
| Facebook person |  |
| Government |  |
| Potential Misconceptions | Participant makes a remark indicating a potential (direct or indirect) misconception regarding DTC-GTs |
| Involved scientists/HCPs | Participant makes a remark that indicates a potential misconception about scientists/HCPs involved with DTC-GT companies |
| Results | Participant makes a potential misconception regarding results of DTC-GT |
| DNA genotype influence by having disease | Participant states that they expect a family's genotype can change if an ancestor has had a certain disease (e.g. genotype changing because some ancestor had the Plague) |
| Possibility of DNA genotype changing | Participant remarks that they deem it possible/feasible that someone's DNA genotype changes over time |
| Impact on insurability | Participant remarks that results of a DTC-GT can have an impact on your insurability or premium of insurance. |
| Impact on getting a mortgage | Participant thinks that DTC-GT result could impact if you can get a mortgage or not |
| False reassurance | Participant makes a remark that indicates DTC-GT potentially caused false reassurance (e.g. 'test says I don't have breast cancer gene, so that's a relief', but the test is a SNP-array) |
| Misinterpretation of results | Participant (directly or indirectly) makes remarks indicating misinterpretation of results (e.g. 'I've got a risk of 0.6x, indicating it's higher than the average', etc.) |
| (Medical) misconceptions unrelated to DTC-GT | Participant makes remark that can be categorized as potential (medical) misconception unrelated to DTC-GT |
| Risks & Opportunities | Participant mentions risks and/or opportunities of DTC-GT (general, not for their own decision making process per se). |
| Opportunities | Participant mentions (a) opportunity(s) of DTC-GTs, but not per se in the context of their own decision making process (e.g. 'cheap', 'increased awareness of genetic disease risk in society', 'decreased burden on healthcare', 'disease prevention', 'decreased healthcare costs' etc.) |
| Risks | Participant mentions (a) risk(s) of DTC-GTs, but not per se in the context of their own decision making process (e.g. 'threatens equity in society', 'risk for unwarranted anxiety', 'risk for receiving unexpected results', 'risk for burden on public healthcare system', etc.) |
| DTC-GT related recommendations by participant | Participant makes remarks concerning recommendations they have in the context of DTC-GT, and stakeholders they deem responsible/capable of implementing/executing these recommendations. E.g. in the context of supporting decision making, making DTC-GT more accessible, protecting consumers against harmful effects of DTC-GT, and incorporation of DTC-GT in the regular healthcare system. |
| Responsible stakeholders | Parties people deem responsible/suitable for developing/enforcing/implementing DTC-GT related decision support & care and/or policy. |
| DTC-GT seller |  |
| Consumer organizations | E.g. Consumentenbond, Kassa, Radar, etc. |
| HCPs |  |
| Regular healthcare |  |
| Alternative healthcare |  |
| Universities |  |
| Scientists |  |
| Hospitals |  |
| Government |  |
| Regular media | Participant names regular media (e.g. newspaper, radio, tv) as a stakeholder for a certain recommendation |
| Recommendations | Topics for which/how participants do recommendations for improvement |
| Relevant pre-test | Consumer does recommendation that is relevant pre-test in the DTC-GT consumer journey (e.g. improving pre-test information provision, increasing awareness, decreasing barriers, 'mandatory' pre-test consult with HCP, etc.) |
| Increasing awareness | Participant states that they find it important that more awareness is created regarding health-related DTC-GT, and/or by which party |
| Decreasing barrier to undergo DTC-GT | Participant remarks that they find it important that the barrier to undergo DTC-GT is lowered |
| Pre-test information provision | Participant remarks whether they feel pre-test information provision regarding DTC-GTs should be improved, and/or by which party |
| 'Mandatory' Pre-test HCP consult | Participant states that they would be in favor of a mandatory pre-test HCP consult to determine if someone should do a DTC-GT, or at least advise them of the risks inherent to doing such a test. |
| Quality marks | Participant mentions the recommendation/wish that there would be DTC-GT quality marks |
| Disclaimers regarding quality/reliability/consequences of DTC-GT | Participant mentions a need for disclaimers about the quality/reliability/potential consequences of DTC-GT |
| Relevant post-test | Consumer does recommendation that is relevant post-test in the DTC-GT consumer journey (e.g. educating HCPs about usability of DTC-GT results, post-test info provision, improving result communication, locking results to sensitive diseases, post-test consultation) |
| Post-test support system | Participant remarks that they would appreciate some form of post-test support system (e.g. counseling options in regular health care, counseling or customer service provided by seller, etc.) |
| Locking results to sensitive diseases | Participant makes remarks regarding the need for manually unlocking certain results (e.g. Alzheimer's, breast cancer risk) and their views thereon |
| Post-test information provision | Participant remarks whether they feel post-test information provision regarding DTC-GTs should be improved (e.g. recommendations for follow-up reading, information about where you can go to get help to interpret your results, etc.), and/or by which party |
| Areas of improvement regarding result communication | Participant remarks areas of improvement concerning the results (e.g. 'show absolute risk rather than relative risk', 'prioritize the ranking of diseases based on disease risk', 'I wish I also had access to XYZ myself, rather than having to get that through someone else', 'It would be good if they added ABC to the test', etc. |
| Educating HCPs | Participant remarks that they find it important that HCPs are educated about DTC-GT and the usefulness of their results |
| Spamming consumers with additional offers | Participant states they would like DTC-GT sellers to not spam them with additional offers after initial consumption of a DTC-GT |
| Integration of DTC-GT results in health records | Participant states that they would like to see option to integrate their DTC-GT results in their health records so that HCPs can take that into account. |
| Relevant beyond consumer journey | Participant does recommendations that are relevant beyond the DTC-GT consumer journey (e.g. generating universal reference DNA databases, generating neonatal DNA passports universally, suggestions for policy such as implementation of regulation and legislation, quality marks, etc.) |
| Public-Private collaborations | Participant states that they would appreciate public-private relationships where data from DTC-GT companies can be used by research or medical decisions within the public healthcare system. |
| Neonatal DNA passport generation | Participant remarks that they are in favor of allowing people to generate a neonatal DNA passport |
| Generation of universal reference DNA databases | Participant suggests development of a universal DNA databases (through combining currently available DNA databases) to enhance reliability of DTC-GT results |
| Yearly 'human MOT' | Participant recommends implementation of a nation-wide yearly 'human MOT' |
| Increased regulation of DTC-GT market | Participant advocates for increased regulation of the DTC-GT market in the Netherlands |
| Analogies/Antagonies with other self-tests | Participant makes remark where they draw analogies/antagonies between DTC-GT and other self-tests (e.g. total body scans) |
