# Supplementary material for: Navigating direct-to-consumer genetic testing: experiences, decisions and perspectives of Dutch users
Source: Eur J Hum Genet. 2026 Feb 4;34(4):480–90. doi: 10.1038/s41431-026-02022-z (PMC13046840; doi:10.1038/s41431-026-02022-z)
Supplement: Supplementary file 3 — Supplementary Material 3: Supporting Quotes [file 41431_2026_2022_MOESM3_ESM.docx]

**Navigating direct-to-consumer genetic testing: experiences, decisions and perspectives of Dutch users**

**European Journal of Human Genetics**

**Author information**

***Danny Bruins^a^, Esther A.M. Bührman^a^, Martina C. Cornel^a^, Margreet G.E.M. Ausems^c^, Marc H.W. van Mil^d,e^, Olga C. Damman^f^, Tessel Rigter^a,b^***

^a^Section Community Genetics, Department of Human Genetics, Amsterdam Public Health Research Institute, Personalized Medicine, Amsterdam UMC, Vrije Universiteit Amsterdam, 1105 AZ Amsterdam, The Netherlands.
^b^Center for Health Protection, National Institute for Public Health and the Environment, 3721 MA Bilthoven, The Netherlands.
^c^Department of Genetics, Division Laboratories, Pharmacy and Biomedical Genetics, University Medical Center Utrecht, 3584 CX Utrecht, The Netherlands.
^d^Center of Education and Training, University Medical Center Utrecht, 3584 CX Utrecht, The Netherlands.
^e^Center for Molecular Medicine, University Medical Center Utrecht, 3584 CX Utrecht, The Netherlands.
^f^Department of Public and Occupational Health, Amsterdam Public Health Research Institute, Quality of Care, Amsterdam UMC, Vrije Universiteit Amsterdam, 1105 AZ Amsterdam, the Netherlands.

**Corresponding Author:** Danny Bruins, d.bruins@amsterdamumc.nl

**Funding:** This study was conducted as part of the ERUDIGIT project, funded by Netherlands Organisation for Health Research and Development, grant number 05550402110010.

Supplementary Materials 3: Supporting Quotes^a^

An extensive table with quotes supporting the descriptive themes outlined in the Results can be found underneath. Selected representative quotes per theme can also be found in Table 2.

| **Theme** | **Quote** | **Quote Number** |
| --- | --- | --- |
| Varying initial contact sources and opinions regarding DTC-GT | *‘Well, I went digging in my own medical history, and yeah, via Facebook I ended up in certain groups, and via there I got into contact with someone that had also done a DTC-GT. They shared their knowledge with me like: ‘It works like this and that and you can get a lot of information out of that.’, so that’s how I ended up there, through their experience.’ (P3, F42, HL+, NUM-, l.80)* | Q1 |
|  | *‘… I literally ran into it in the waiting room of the hospital. Someone that participated in the same research project as I was participating in, told me like ‘there’s tests that can give you insights about what to focus on, like whether you are at increased risk of cardiovascular disease and diabetes and stuff like that.’ And then he gave me that brand. I wrote that down in my phone and ordered it later.’ (P10, M40, HL+, NUM+, l.22-26)* | Q2 |
|  | *‘Well actually, I first came into contact with it via my GP… She is also an orthomolecular doctor, so outside of her GP practice, she has a consultation hour on Saturdays for patients that are interested in that [orthomolecular therapy]… and then she talked about those DNA-tests so that you can see on DNA-level what your body needs to perform its functions better… So that’s why I did that.’ (P18, F72, HL+, NUM-, l.14-28)* | Q3 |
|  | *‘I think I heard of it first via regular media, I suspect via the radio.’ (P4, M64, HL+, NUM+, l.72)* | Q4 |
|  | *‘[When I heard about health-related DTC-GT for the first time it was] eye-opening. I was thinking: ‘Wow, amazing that this is available in the Netherlands!’, I thought that was all American type of stuff, so I was very happy I could do it too!’ (P1, F59, HL+, NUM-, l.41-46)* | Q5 |
|  | *‘… And after I learned more and more about it, about the quantity of information it could yield, I was thinking: ‘Well, it would be very interesting to also do that myself!’… I was thinking: ‘I want to do that too.’.’ (P5, M54, HL+, NUM-, l.32)* | Q6 |
|  | *‘Well look, it is a commercial company, everyone has their own interests and a commercial company like a DTC-GT seller… Then I am wondering: ‘What is your incentive?’ And of course it is your DNA that you are giving them. And yeah, with data leaks and such, I do not want my DNA to end up anywhere in the world… So yeah, I was very skeptical at first, like I said because it is commercial and such.’ (P3, F42, HL+, NUM-, l.92-110)* | Q7 |
|  | *‘Yeah, I wasn’t taking it [health-related DTC-GT] very seriously at first, because I was thinking: ‘There’s no way that can work.’.’ (P14, F38, HL+, NUM-, l. 154)* | Q8 |
| Pre-test information acquisition | *‘Well, for me it was very important to know where I could find good information about what enzyme mutations can mean… and whether I could get my DNA off of that platform… I got that via that woman that I spoke to via Facebook, she has an enormous amount of experience with this… She really dove into this stuff.’ (P3, F42, HL+, NUM-, l.150-160)* | Q9 |
|  | *‘Well, I also looked at experiences of previous consumers… If more than half of those are negative, there’s always negative ones, but there mainly have to be positive ones. So I looked at that to see whether it’s really reliable. But based on the reviews and explanation on the [seller’s] website and stuff, yeah I had a good feeling about it.’ (P8, F40, HL+, NUM-, l.88)* | Q10 |
|  | *‘It was via the website of [name of orthomolecular therapist], underneath the heading [name of DTC-GT seller]. There it was clearly explained how it worked… There was a long explanation there.’ (P1, F59, HL+, NUM-, l.178)* | Q11 |
|  | *‘Initially, I’ll have looked into information from the Consumentenbond [Dutch consumer organization]… I’m a member there, they’re always my first go-to source of information, because you’d assume they’re objective.’ (P4, M64, HL+, NUM+, l.56-58)* | Q12 |
|  | *‘Yeah, from websites where they compared those things [DTC-GTs from different sellers] with each other. I looked at which traits they tested for, how extensive their test is so to say. And I think I also looked at how confidential they are and, yeah, the methods that they use… And then I started reading about which are reliable, based on customer reviews and such.’ (P16, F30, HL+, NUM+, l.55-60)* | Q13 |
| Pre-test expectations: hopes, actions and consequences | *‘Uh, yeah, [I expected that] I would get a lot of information that I could use to adapt my lifestyle. Checking if what we’re doing now is good or whether we’re missing certain things, because we’re predisposed to something we’re not aware of. Checking if we had to finetune some stuff.’ (P5, M54, HL+, NUM-, l.170-172)* | Q14 |
|  | *‘Well, I was very curious about the drug sensitivity part, and whether something handy would come out of that that I could utilize.’ (P7, F55, HL+, NUM-, l.192)* | Q15 |
|  | *‘I think I was expecting risk estimates for certain diseases, and how you can deal with that.’ (P17, M52, HL-, NUM+, l.96)* | Q16 |
|  | *‘Insights into what you would have to get investigated further, like: ‘If there’s an increased risk… get this and that and that explored in-depth.’ For example with cardiovascular disease: ‘get a scan done that visualizes certain veins.’.’ (P10, M40, HL+, NUM+, l.134)* | Q17 |
|  | *‘Well, I was expecting something from it regarding my health and how to improve that… Feeling better in your own body through taking the supplements that are right for you… That was my goal [with the test].’ (P18, F72, HL+, NUM-, l.92-104)* | Q18 |
|  | *‘I’m very much a fan of knowing things. For example, If I know something, I’d be able to draw up a will, or warn my kids about it. If it says something about your future, it’s handy [to know].’ (P6, F61, HL+, NUM+, l.668)* | Q19 |
|  | *‘… Maybe ‘curiosity’ doesn’t fully cover it, it might be a bit too thin, but I wouldn’t know how to express it any other way. It wasn’t like I had anything like: ‘Oh, maybe I’ve got this’, or ‘There’s high cholesterol, cardiovascular problems, brain problems in different family branches’, no.’ (P4, M64, HL+, NUM+, l.208-210)* | Q20 |
|  | *‘… It wasn’t that I was feeling miserable or something. No, I just thought it was fun and wanted to get to know myself better. One person does that in their heads, others want to get that on paper.’ (P9, M50, HL+, NUM-, l.54)* | Q21 |
|  | *‘Expectations? I’d rather say hope. I was running into so much lack of understanding of my vague complaints within the regular healthcare system that I hoped to find something that would stop the constant discussion, that’s how you could summarize it.’ (P3, F42, HL+, NUM-, l.226)* | Q22 |
|  | *‘I expected that the test would tell me I had Pompe disease, I was convinced of that… I expected something concrete to come out of the test immediately.’ (P6, F61, HL+, NUM+, l.432)* | Q23 |
|  | *‘Well, I’ve been having health complaints for years, especially with my weight that I just can’t seem to lose, and then you start thinking like, you’ll search for anything that you might be able to use… And also because cardiovascular disease runs in my family and I often have complaints related to that, but they couldn’t find anything within the public healthcare system, so I was thinking: ‘I’ll check whether it’s hereditary or not, so that I can keep that in mind’, or something like that.’ (P8, F40, HL+, NUM-, l.26-31)* | Q24 |
|  | *‘Well before I became pregnant, this was actually some type of screening for that like: ‘Is it wise to do that?’… Look, I can be carrier of something that doesn’t come to expression for me, and the same can be the case for my partner, things that don’t affect us, but imagine we both pass it on, then our child could be affected. So that was important to learn: what are we carriers for that we don’t know?’ (P12, F42, HL+, NUM+, l.122-124)* | Q25 |
|  | *‘So I just wanted to know whether I for example was carrying a gene that I could pass on if I ever had kids that could be damaging, stuff like that.’ (P16, F30, HL+, NUM+, l.10)* | Q26 |
|  | *‘I felt like I had very little choice, I wanted to know before I would have had kids… So that I could tell my future partner like ‘I did this test, it says I’m a carrier of cystic fibrosis, I want to get that officially tested via the hospital so that you can get tested as well, to ensure our kid will not have cystic fibrosis.’.’ (P19, F23, HL+, NUM+, l.78)* | Q27 |
|  | *‘Well, I was expecting to learn whether I had Chinese or Uruzganian blood or whatever. Or the percentages of where I’m from, whether it was only North-European, or Danish or Swedish or I don’t know, Scandinavian. [Interviewer: and what about the health module?] That was a byproduct. I wasn’t expecting anything from that at all.’ (P9, M50, HL+, NUM-, l. 266-272)* | Q28 |
|  | *‘That [health module] was a byproduct, yeah.’ (P2, M70, HL+, NUM+, l.112)* | Q29 |
|  | *‘Well, if you have something very severe, it might be nice to know in advance, but it also might not be nice to know… I think that it could cause some people to lose their minds like: ‘Well, I can’t be saved anymore, so never mind.’.’ (P11, F68, HL+, NUM+, l.24-26)* | Q30 |
|  | *‘I’d never want to be able to look into the future, so imagine that something terrible would happen, I would not want to know that I’m dying next week. So that gets you thinking like: ‘What am I getting myself into?’. Imagine that something comes out of it that you can’t act upon, it might have been better to just bury your head in the sand.’ (P20, F54, HL+, NUM-, l.70)* | Q31 |
|  | *‘For example, my son told me: ‘If I commit a murder, they can find out via your DNA that I did that.’… Or for example if a cousin of mine turns out to be a criminal or involved in something [criminal].’ (P9, M50, HL+, NUM-, l.62-66)* | Q32 |
|  | *‘Yeah, and I did warn my kids that I was going to do this and that I was doing one that was very good with privacy, but that you never know for sure, so if their DNA ever ended up on a crime scene somewhere, that I couldn’t do anything about it, but my DNA sat somewhere in the system.’ (P6, F61, HL+, NUM+, l.298)* | Q33 |
|  | *‘But I can imagine that some people might not want to do it, because you may have trouble with insurances and such. Good luck getting disability insurance if you have high risk of pancreatic cancer. Well, that price is going to skyrocket of course.’ (P1, F59, HL+, NUM-, l.166-170)* | Q34 |
|  | *‘Well, if you know that you’re at increased risk for certain diseases, that could of course influence whether or not you’ll be able to get a mortgage, or maybe even life insurance.’ (P7, F55, HL+, NUM-, l.200)* | Q35 |
| Arguments driving decision-making | *‘Well the price, it was cheap and, uh, the convenience. You’re getting it sent to your home, you don’t have to leave your house.’ (P10, M40, HL+, NUM+, l.86)* | Q36 |
|  | *‘And yeah, for only a small extra price, you could also order the medical insights [in addition to heritage-related insights]. So I thought that over and then I decided to do that too… I waited to order until there was a sale, there’s one every so often.’ (P4, M65, HL+, NUM+, l.10 and l.26)* | Q37 |
|  | *‘I knew that they existed, these tests, and then all of a sudden you see an ad that says ‘Now on sale!’, and I’m thinking: ‘Well, why not, I’ll do it’… I was already interested, and that won me over.’ (P2, M70, HL+, NUM+, l.214-220).* | Q38 |
|  | *‘The biggest objection for me was the price… and then at a given moment there was a sale. They run sales every so often and then I thought: ‘I’m doing it now!’. I think they normally cost $400, maybe $500. They were really doing price stunts back then.’ (P6, F61, HL+, NUM-, l.296-298)* | Q39 |
|  | *‘Every so often there’s a sale, and then you can temporarily use it for free.’ (P14, F38, HL+, NUM-, l.12)* | Q40 |
|  | *‘Well, I have a friend that did the DTC-GT first. That friend was very positive about it… And my friend’s partner is a chemist, whom was really impressed by how it worked. And that person is not impressed quickly… That chemist knows his stuff, so he also understands that what’s being sold, is not some nonsense.’ (P1, F54, HL+, NUM-, l.14 and l.196-200)* | Q41 |
|  | *‘Recommendation by an acquaintance. That’s someone that gets to the bottom of things, and the odds of them doing something nonsensical is negligible. They always study stuff well. (P5, M54, HL+, NUM-, l.96-98)* | Q42 |
|  | *‘I think that my partner and I were convinced to do the test by the experiences of a family member.’ (P17, M52, HL-, NUM+, l.16)* | Q43 |
|  | *‘My family member is a perfectionist, she looks up everything, and so she ended up at this test… She did it, and was very enthusiastic about it. My family member’s partner did it as well… And like I said, my family member is a perfectionist, looks up everything. I do so as well, but if she says something, I trust her.’ (P20, F54, HL+, NUM-, l.14 and l.78)* | Q44 |
|  | *‘Just to do it together with my partner, uh, to both have experienced it to be able to talk and discuss about it… being a conversational partner in a sense.’ (P5, M54, HL+, NUM-, l.66-68)* | Q45 |
|  | *‘Well, I’ve been taking care of my partner for over two years now due to medical reasons. So I approached my orthomolecular therapist, explained what was going on, and he advised to do such a test, because that might help to discover something that could help… My partner didn’t want it at all, but in the end, we both did it… I personally did it mainly out of curiosity, but also to help persuade my partner to do the test.’ (P13, F71, HL+, NUM+, l.18 and l.68)* | Q46 |
|  | *‘And it’s easy of course. We didn’t have to do very complicated stuff. It’s a matter of sending it and then just waiting.’ (P1, F59, HL+, NUM-, l.518-520)* | Q47 |
|  | *‘And I also run an orthomolecular practice myself, so from that viewpoint I was interested in what these tests could do. So that’s why I bought one. I was thinking: ‘Imagine I want to start advising these to people, than I have to know what they entail myself’. So that’s why I did it.’ (P7, F55, HL+, NUM-, l.14-18).* | Q48 |
|  | *‘[Interviewer: ‘So were there any possible reasons you considered to maybe not undergo the test, or that made you doubt that’?] No, I’m rather impulsive with a lot of stuff… I didn’t really consider like ‘what if…’, I saw the opportunity and decided to do it.’ (P9, M50, HL+, NUM-, l.90)* | Q49 |
|  | *‘Being brutally honest, I went into this really impulsively and didn’t really read into it at all.’ (P12, F42, HL+, NUM+, l.128)* | Q50 |
|  | *‘[Interviewer: ‘Were there any possible for you to not do this test, or that made you doubt doing it?’] No, not at all.’ (P8, F40, HL+, NUM-, l.44)* | Q51 |
|  | *‘I decided to go with this firm because it had a privacy-story that seemed most reliable to me. I wouldn’t be able to reproduce it, but it was all very… I believe you wouldn’t even be able to get to your DNA data if you had forgotten your code, something like that. Also that they would never give anything to the FBI… I thought it really sounded like they found that important, and really put thought into it.’ (P6, F61, HL+, NUM+, l.232-234)* | Q52 |
|  | *‘Well, the main thing that went around in the back of my head was privacy, like what happens with your data… You’re giving them your data, so to say. They can say that it’s anonymous, but what is ‘anonymous’? I mean, Facebook should be anonymous as well, but you’re still monitored to some degree, so to say. That gave me some doubts.’ (P15, M41, HL+, NUM+, l.32-34)* | Q53 |
|  | *‘Well I was definitely considering my privacy, that there is potential for misuse, that the data can end up at your health insurer or something like that.’ (P16, F30, HL+, NUM+, l.24)* | Q54 |
|  | *‘GDPR, I found that very important, the privacy. I find it very important that stuff that’s mine, stays mine… I always check who is behind a company and to what degree they can guarantee my privacy… So things like: what happens to my DNA that’s attached to my personal data? Won’t it end up somewhere in the world with the highest bidder? What happens if there’s a data leak?’ (P20, F54, HL+, NUM-, l.46)* | Q55 |
|  | *‘But yeah, it’s the costs that are attached to it that make you think like: ‘That’s almost €200!’, you know?’ (P8, F40, HL+, NUM-, l.18)* | Q56 |
|  | *‘Well, the costs were rather steep.’ (P16, F30, HL+, NUM+, l.38)* | Q57 |
|  | *‘It’s rather costly… It’s a lot of money, you’re wondering what you’re going to even get out of it.’ (P17, M52, HL-, NUM+, l.22)* | Q58 |
|  | *‘I did want to take this seriously, because if you get bad news, you have to be able to deal with that. It has to be a conscious decision that you’re making. You shouldn’t do this in an impulse.’ (P4, M64, HL+, NUM+, l.268)* | Q59 |
|  | *‘Imagine I would have the BRCA-gene, would I be able to get a mortgage? Or would an insurer say: ‘I’m going to exclude you.’?’ (P20, F54, HL+, NUM-, l.46)* | Q60 |
|  | *‘I did talk about it yes. Definitely with my partner. But they know me, and if I’ve decided I’m going to do something, I’ll go and do it… I also think I discussed it with friends. But that’s an exchange of thoughts. No one is going to tell me what to do or not do… All these opinions and considerations are weighed. I listen to people, but I won’t always do as they say. The consequences are for me to bear, so I have to make that decision.’ (P4, M64, HL+, NUM+, l.290-298)* | Q61 |
|  | *‘I did discuss with my partner, of course it’s not just something you decide to do on a whim… And also with my parents… [Interviewer: ‘To what degree did those conversations play a meaningful role in your decision-making process?’] They most definitely did. That goes for this, but also other important stuff. I always consult my partner and parents then. That isn’t to say it would immediately make me say ‘I’m not going to do it’ if they’re not positive though, no.’ (P15, M41, HL+, NUM+, l.38 and l.110-128)* | Q62 |
|  | *‘[Interviewer: ‘You mentioned you discussed undergoing this test with your partner multiple times. To what degree did those conversations play an important role in your decision-making process?’] They definitely did, yes. [Interviewer: ‘Imagine your partner would have had a more negative attitude towards these tests than they did, would you still have gone through with it?’] It would depend a bit on the reason why they would be negative. Uhm, I think that, in that case, I wouldn’t have gone through with it.’ (P17, M52, HL-, NUM+, l.83-94)* | Q63 |
|  | *‘…He [orthomolecular therapist] has helped me a lot in the past. I have very good experiences with him, I know him, so I thought like: ‘If he’s recommending it, we’re going to try it’.’ (P13, F71, HL+, NUM+, l.30)* | Q64 |
|  | *‘…I even suspect I told my GP that I was considering this, but I don’t remember how she reacted to it. But if she would’ve strongly advised not to do it, I really wouldn’t have done it.’ (P4, M64, HL+, NUM+, l.708)* | Q65 |
| Consumers’ pathways and decision timelines | *‘Actually, I think I decided to do it immediately, maybe thought for an hour, but no more than that.’ (P11, F68, HL+, NUM+, l.42)* | Q66 |
|  | *‘No, no, no I did not immediately do it. I really thought about it for a long time. I think like a year or something.’ (P3, F42, HL+, NUM-, l.134)* | Q67 |
| Post-test evaluation of test findings and insights | *‘No, actually, yeah your attention is drawn to those things that say ‘increased risk’… Yeah that grabs your attention first.’ (P3, F42, HL+, NUM-, l.210)* | Q68 |
|  | *‘Well, if you open the report, the most impactful things are listed at the top, and if it’s green, it’s positive, and if something’s negative it’s red.’ (P12, F42, HL+, NUM+, l.236)* | Q69 |
|  | *‘…And especially with that color coding, that makes you check out the orange [high-risk] things first.’ (P18, F72, HL+, NUM-, l.260)* | Q70 |
|  | *‘Well, I don’t know that off the top of my head, I can read them for you though. So I’ll read them from the app… So indeed, you get an overview of your risks for diseases and lifestyle recommendations corresponding to that… Also some other personal characteristics. The best thing is that it confirmed I’m male [laughs]. And in addition to that you receive an overview of drug sensitivities, and something with a pharmacological profile.’ (P17, M52, HL-, NUM+, l.144-150)* | Q71 |
|  | *‘Yeah, you get an overview with like 6 boxes, which show you ‘genetic predisposition to disease’, ‘hereditary monogenic diseases’, ‘pharmacological compatibility’, ‘personal characteristics’, ‘wellness’ and ‘ancestors’. You can then click one of those boxes and then you’ll see what type of stuff they’ve tested and which spots [in the genome] they’ve tested. And then there’s a diagram for example with ‘high, low, average’ and then it’s indicated on that line where your risk lies.’ (P16, F30, HL+, NUM+, l.134)* | Q72 |
|  | *‘No, you really have to dive into that every once in a while, because it isn’t easy to understand. You have to read a lot about what it entails. I’ve saved everything to a hard-drive and I just read, read, read. Scientific articles, information that the TPI gives, research, yeah you’re reading all of that. It’s not like: ‘Oh, I’ll just upload it there and I’ll know what’s up’, no. I’ve put two years into it.’ (P3, F42, HL+, NUM-, l.406-414).* | Q73 |
|  | *‘…I had not anticipated the amount of work it would be to be able to understand it [the results]… I think I’ve spent a year puzzling with the results before I understood how to approach it.’ (P6, F61, HL+, NUM+, l.460)* | Q74 |
|  | *‘But look, it’s got a very large learning curve so to say, because if you read such a report, it’s very scientific, lots of specific terms. It isn’t something for a standard user, so to say, you really need to dive into things like how to interpret the results and find out what they mean. It isn’t something everyone should do without thought.’ (P12, F42, HL+, NUM+, l.248)* | Q75 |
|  | *‘Well, it’s easy to read. It isn’t explained in layman terms, but it’s also not medicalese… No but look, it’s neat isn’t it? I think that, of course you’re looking at it from your own point of view, but I feel like the average Dutch person should be able to read this right? I feel that this is accessible for the average Dutch person.’ (P1, F59, HL+, NUM-, l.694-700)* | Q76 |
|  | *‘And uh, I did have to really read in-depth about what it said and the result, how I should interpret that. For example, here in the app it says: ‘Alzheimer’s disease’ and then it says: ‘lowered risk of Alzheimer’s disease’ with a mini-graph and the number ‘0.5x’. Well that makes you think: ‘That’s okay!’, but if you then tap on that in the app, it says ‘risk is halved compared to the average’. And then I thought like: ‘Oh, so that’s how I’m supposed to read that’. It’s important to realize that… At first I didn’t really find it very intuitive.’ (P17, M52, HL-, NUM+, l.156 and l.198)* | Q77 |
|  | *‘Even a bit of reference like, uh, are those tests really correct? Like, do we find overlapping stuff that’s correct, or overlapping stuff that isn’t correct? That’s after the fact, but it has me thinking like: ‘Let’s compare.’ and we just see so many things that match up, that they couldn’t have known in advance… it makes you become more confident of it. Not that I doubted that in advance, but you always want to check that in some way or another.’ (P5, M54, HL+, NUM-, l.72-82)* | Q78 |
|  | *‘Yeah, it seemed reliable… because I recognized myself in certain results. It made the results make sense. I would’ve had doubts if they [the results] would’ve been very different.’ (P15, M41, HL+, NUM+, l.254-258)* | Q79 |
|  | *‘I don’t expect that the markers that they look at, that they knowingly assess them wrongly. I mean, if you’ve got a certain code on something, then that’s the case. What reason would them have to do that differently?’ (P4, M64, HL+, NUM+, l.94-98)* | Q80 |
|  | *‘Yeah, yeah I think it’s reliable, because why would they put wrong stuff in there [the report]?’ (P11, F68, HL+, NUM+, l.186)* | Q81 |
|  | *‘Well, I had faith in it, because it was recommended by my orthomolecular therapist.’ (P13, F71, HL+, NUM+, l.304)* | Q82 |
|  | *…Yeah, and the fact that my GP recommended it, I trust in them as well of course.’ (P18, F72, HL+, NUM-, l.322)* | Q83 |
|  | *‘I’m taking it with a large grain of salt… because you don’t just have those diseases, but also here for example: ‘80% chance that you have no to little hair on your back’, well, my partner sometimes calls me a silverback gorilla, so yeah [laughs], I’ve got more hair on my back than on my head… ‘77% chance you’ve never had dandruff’, well I did have that. When you look at stuff like that, it makes you think it’s still a bit of guesswork… All those traits, if I look at those, most of which are only so-so correct, how serious do I have to take that medical test then?’ (P2, M70, HL+, NUM+, l.128-160)* | Q84 |
|  | *And of course, I don’t know how reliable it all is. You hear very different stories about that in the media… So then you read about a consumer organization that made an item about that, and that you don’t actually know what happens to your data, and whether it’s reliable or not.’ (P9, M50, HL+, NUM-, l.54-60)* | Q85 |
|  | *‘Well look, I can remember going to my GP afterwards, because I had an increased risk of diabetes [according to the test]. So I went to the GP, and the GP thought the test was ‘bogus’, ‘controversial’. He said: ‘Yeah, this is just a money grab of sorts.’… I felt screwed actually, because when I heard that, I thought: ‘That’s been a waste of money.’.’ (P10, M40, HL+, NUM+, l.50-52)* | Q86 |
|  | *‘So yeah, now I’m actually starting to wonder: is it all true, or is it all about the money they’re trying to rake in?’ (P11, F68, HL+, NUM+, l.204)* | Q87 |
| Post-test information acquisition | *‘Via PubMed I ended up at that pharmacist, he put me into contact with someone else, who put me into contact with yet someone else, who brought me into contact with the professor. So all of that goes via a single publication on PubMed.’ (P1, F59, HL+, NUM-, l.760)* | Q88 |
|  | *‘Well, you’ve got this, ‘Layman’s overview’, so to say, and they’ve also got a ‘Scientific Explanation’. And that’s a bit more in-depth, but yeah, to properly judge that, you need some more in-depth knowledge. I don’t have that. I didn’t look at that really deeply… I just looked at the overview, and I think that’s what most people do.’ (P2, M70, HL+, NUM+, l.406-418)* | Q89 |
|  | *‘I mean, my partner and I, altogether it was €1.000, €500 for my partner, and €500 for me… For €250 you only get the genotype and then during an orthomolecular consult, you can ask the therapist to open 7 additional boxes. And if one of those boxes is opened, the therapist has to pay an amount to the seller. So you see the number [price] going up. So let’s say I want to know something about estrogen, well, there goes €70. Glutathione, €30, etcetera… No, you can’t open those modules yourself. He has a key to unlock those, the seller thinks it’s too dangerous to provide those data without expert advice.’ (P1, F59, HL+, NUM-, l.210-224)* | Q90 |
|  | *‘…If you do the comprehensive DTC-GT, you only get half of the information after consultation with an expert of the company, they don’t give you that information without any explanation. We got that information and then they’ll dive even deeper into the processes and abnormalities… That specialist quickly asked ‘Aren’t you suffering from headaches?’ I said ‘No’, but by that time it was so regular for me to suffer from headaches, so I said ‘Oh, yeah, yeah’, and he said: ‘Yeah, because I can see here very clearly, that’s so remarkable, that you process histamine very badly, so large chance that that’ll make you suffer from headaches.’ So he said ‘that’s due to this and that, you’re missing that enzyme, if you supplement that in supplement form, you should notice that’. Well, that was the case.’ (P5, M54, HL+, NUM-, l.214)* | Q91 |
|  | *‘Well, there’s a couple of, uh, you get a list of doctors that can give you a consult based on these tests, so interpreting your results and inform you more about that.’ (P17, M52, HL-, NUM+, l.30)* | Q92 |
|  | *‘…I did write down an address, because it did say that if you ordered the comprehensive package, you could, for additional payment of course, then there was some doctor in my neighborhood, you could go through the enzyme-list with them and they could give advice about diet and such based on the outcomes of that list.’ (P8, F40, HL+, NUM-, l.318)* | Q93 |
|  | *‘Uhm, no, let me think, what was it again? The therapist didn’t sell them [the supplements he recommended], but they did give me some kind of coupon for ordering them at some store online… So yeah, the therapist will probably get a percentage from those sales, but oh well, yeah… There’s probably a revenue model for the therapist there’ (P17, M52, HL-, NUM+, l.302-306)* | Q94 |
|  | *‘I’m not a fan of statistics, I want personalized medicine… If you’re being undertreated, you’re taking that trash [cancer drugs] for nothing, and you’ll still die eventually. That’s not the intention of course.’ (P1, F59, HL+, NUM-, l.94-96)* | Q95 |
|  | *‘So now I had an argument to get targeted exploration of disease risk at my GP, because I’m seeing ‘increased risk’, so I just want to know what’s up.’ (P10, M40, HL+, NUM+, l.72)* | Q96 |
|  | *‘…So then I went searching for all sorts of genes involved in muscle disorders… and put all of those genes in that program that I just showed you, and all of a sudden one popped up in which I had two mutations that were not OK… So when I found those two mutations, I told my cardiologist, and he said: ‘It is important that we look into that a bit more’, and referred me to the clinical genetics center. There they said: ‘Yeah, two mutations…’, and one was proper bad, that was known, but the other was unknown, but it could very well be bad, so to say, it was located in a not-unimportant part of the gene… So yeah, there’s a large chance that my muscle problems are due to this gene… The conclusion was that you can’t be sure, but I was advised to be careful with it.’ (P6, F61, HL+, NUM+, l.470-516)* | Q97 |
|  | *‘Initially, the oncologist wouldn’t go along with it [the DTC-GT results for drug sensitivity]. He said: ‘That’s nice and all, but we’re not going to do that. The standard dosage is 20 milligrams, so you’re getting 20 milligrams, now get out.’… Eventually I ended up at another hospital, and they gave me 30 milligrams after some measurements.’ (P1, F59, HL+, NUM-, l.112-128)* | Q98 |
|  | *‘Yeah, I know multiple people that got tested… Some stuff came out of that with which they went to the GP, who was not very receptive to that… He didn’t want to do anything with it initially… He wasn’t very accommodating.’ (P12, F42, HL+, NUM+, l.166-168)* | Q99 |
|  | *‘Because some results from my own research showed risks for medicine usage. And every time I kept getting refused for follow-up testing and then I said: ‘If I don’t get a referral and no pharmacogenetic testing’, because my own research wasn’t being taken seriously in the public healthcare system, ‘you’ll be responsible if it goes wrong.’, because it had already gone wrong in the past, which left me with this speaking disorder. After that, I got the referral.’ (P3, F42, HL+, NUM-, l.22)* | Q100 |
|  | *‘You know, I would actually like to do that [share DTC-GT results with GP], but I also thought: ‘I don’t want to waste those people’s time by sharing this with them now.’.’ (P16, F30, HL+, NUM+, l.236)* | Q101 |
|  | *‘Look, if they just use a single genetic marker [for disease risk estimation], I’d almost say: ‘Look at yourself and just shrug your shoulders.’, if you get what I mean. That doesn’t say anything… Also, we’re talking about increased sensitivity to gluten here, not about heightened cholesterol or cardiovascular disease risk or something. If that were the case, I would’ve presented it to my GP.’ (P4, M64, HL+, NUM+, l.512-522)* | Q102 |
| Health-related actions and experienced impacts | *‘Well, due to those supplements, there was one that I’d never heard of before, and it turns out from my DNA that I have very high stress levels, which I wasn’t even aware of myself… The therapist got that out of my DNA and I got a certain supplement… which made me feel an awful lot better.’ (P18, F72, HL+, NUM-, l.180)* | Q103 |
|  | *‘I always say: ‘I started changing my diet since 2014.’… That gave me a lot of energy… And that also fits some stuff that comes out of the DNA-data, so that makes me think: ‘Oh, so that’s quite OK.’, it’s a confirmation of sorts.’ (P7, F55, HL+, NUM-, l.350-352)* | Q104 |
|  | *‘Slightly increased risks of late-onset Alzheimer’s disease… So yeah, that was a piece of information that made me go like: ‘Well alright, good to know.’, it’s not something you can do anything about. You either get it or you don’t. I can’t start taking three aspirins and paracetamol and that cures it, that doesn’t happen… Unless starting tomorrow we know how to solve Alzheimer’s disease, then we can do something about it, but that’s not the case.’ (P2, M70, HL+, NUM+, l.384 and l.426-434)* | Q105 |
|  | *‘I’ve started reaching an age where there’s little you can do anymore. Imagine you’ve got an increased risk of myocardial infarction, then I’m already too late, I should’ve started living more healthily earlier.’ (P4, M64, HL+, NUM+, l.176)* | Q106 |
|  | *‘I have to say those [lifestyle tips] were very general, having me thinking like: ‘You hear those everywhere.’, you know, ‘move a lot, eat healthily’… I thought that was rather general… That’s the standard stuff that everyone knows. That wasn’t very useful to me generally speaking.’ (P8, F40, HL+, NUM-, l.188-192)* | Q107 |
|  | *‘Actually, for all my life I’ve been reacting severely to medication, and that was never taken seriously… I have since developed severe neurological symptoms, wasn’t able to walk or talk anymore. Also eating, I started suffering from attacks of shortness of breath. So I went digging in my medical history for my prior health issues to find why I was suffering from these things, and then I ended up at enzyme-level testing… But I didn’t get that [in the public healthcare system]… So then I did it myself, but that wasn’t taken seriously either in the public healthcare system, because I did it myself. Then I stopped taking my prescribed asthma inhalers, changed around my diet and supplementation, and I got rid of those attacks of shortness of breath… So then I started playing it hard, and said: ‘I’m not trying anything else, I want such a test [enzyme-level testing that is acknowledged in public healthcare system] first, and if we don’t do that, I’ll hold you all accountable for the consequences.’ (P3, F42, HL+, NUM-, l.30-54)* | Q108 |
|  | *‘…What I did do at the time of the COVID-19 pandemic, is check if I was at high risk of getting very sick from that, to decide whether or not I should get vaccinated… My mother and I decided not to get vaccinated. No, we were already having our doubts at the time and then the DTC-GT showed that we were at low risk of getting very ill from it… So we took a gamble and didn’t get vaccinated.’ (P14, F38, HL+, NUM-, l.18 and l.76)* | Q109 |
|  | *‘The BRCA1- and BRCA2-genes, so breast cancer genes, have also been tested, and well, since I’ve hit that jackpot [breast cancer] two times, maybe I’ve got that gene. But I don’t, so that’s a relief. Not that it matters, but it’s nice to know… It’s just pure bad luck.’ (P1, F59, HL+, NUM-, l.100-104)* | Q110 |
|  | *Well, lately, I was really suffering from that [complaints potentially related to cardiovascular disease], having certain palpitations and I even had an ambulance come pick me up one time… My grandfather back in the day, when he had his first cardiac arrest, he was 43 and I’m 40 now. An uncle from the same side of the family, he was almost 50, but had already had angioplasty and such… But apparently that’s not hereditary, there’s no predisposition in my DNA. So that was a relief.’ (P8, F40, HL+, NUM-, l.78)* | Q111 |
|  | *‘I mean, it’s not a walk in the park, and when I discovered that increased Alzheimer’s disease risk, I struggled with that for a while… The first time I opened the report, it said very large and loud in the first two blocks ‘Alzheimer’s disease’ in red. So that had me thinking like: ‘Whoops.’.’ (P12, F42, HL+, NUM+, l.132 and l.288)* | Q112 |
|  | *‘Someone that I know had, which one was that again, oh, ‘cardiovascular disease’, he had like 85% [risk] there. He was really shocked by that.’ (P5, M54, HL+, NUM-, l.300-302)* | Q113 |
|  | *‘Well of course I had been thinking, for example there’s a lot of cancer in my family, because of course you can search endlessly in your DNA and also encounter stuff that can scare you… So it did show an increased risk for certain cancers. I also had that investigated here [in the clinical genetics center], but because it’s another piece of the gene that’s apparently not studied in the correct way [with the DTC-GT], those results didn’t correspond.’ (P3, F42, HL+, NUM-, l.236)* | Q114 |
| Sharing experiences and recommending testing to others | *‘Actually, I only told my dad like: ‘Oh you know, that stuff with grandpa, that’s not hereditary for me.’, that’s what I said. That’s actually the only thing I told about it. Well, and I did talk about it at home, but only in passing.’ (P8, F40, HL+, NUM-, l.406-408)* | Q115 |
|  | *‘Yeah, I definitely shared my enthusiasm with others. For example, at the hair salon, you talk about it and then that lady also turns out to be suffering from something, then I say: ‘You could consider doing such a test, it could yield you some information that could help you.’, so yeah I’m quite enthusiastic about sharing. If I hear people talking about certain problems, I say: ‘Such a test could help you if you would want that.’.’ (P5, M54, HL+, NUM-, l.440-442)* | Q116 |
|  | *‘Yeah, I did recommend it to friends and family, like: ‘You’re all being so negative, go and try it for yourself then!’, when I was at a birthday.’ (P15, M41, HL+, NUM+, l.262-264)* | Q117 |
|  | *‘I will never say to someone that they should or shouldn’t do something… because I always let everyone make their own decisions, because the consequences are also for them. And I would most definitely also discuss the risks if they ask me about that, for example about being aware of results that could be shocking… Be aware that you may get results that you may not want to get.’ (P4, M64, HL+, NUM+, l.684-688)* | Q118 |
|  | *‘Well, I’m not one to quickly recommend something to others. At most I would say: ‘Be aware, these are possible benefits, these are the possible risks, do whatever you want.’.’ (P11, F68, HL+, NUM+, l.278)* | Q119 |
|  | *‘Well, I wouldn’t advise it to everyone. Only to the people of whom I know that they’re capable of interpreting the results. I especially think the TPI isn’t for everyone… You need to be able to understand it.’ (P12, F42, HL+, NUM+, l.414-418)* | Q120 |
|  | *‘It’s very complicated… Yeah, in the present form [TPI], it isn’t suitable for everyone. It also cost me a lot of time and effort.’ (P6, F61, HL+, NUM+, l.876-878)* | Q121 |
|  | *‘Well, I sometimes get that question, and then I also warn them like: ‘You really need to dive into it and read a lot, read scientific articles.’, sometimes it’s very complicated language, you have to want that, and if you don’t, I wouldn’t recommend taking the path that I did… So yeah, will I recommend it? I’m not going to shout it off the rooftops, no.’ (P3, F42, HL+, NUM-, l.542-544)* | Q122 |
|  | *‘Well, like I said, one company gave a scratch card in their Christmas gift boxes, the other gave a DNA-test. I thought that was very original, I thought it would be fun… It gives interaction within the company… When I did it [the DTC-GT] myself, I would have, I thought that [giving a DTC-GT as Christmas present to employees] would be fun to do. And maybe in my enthusiasm wouldn’t have thought about people potentially finding it not so much fun.’ (P9, M50, HL+, NUM-, l.648-654)* | Q123 |
|  | *‘[Interviewer: ‘So you told me you shared your results and experiences with others’. Are you aware of them also potentially doing such a test themselves?’] Well, three of them at least. They all did such a test. [Interviewer: ‘Based on your recommendations?’] Yes.’ (P1, F59, HL+, NUM-, l.409-418)* | Q124 |
|  | *‘Yeah, and the reason why I also had my child tested is purely rational, you can do a lot with prevention, so I thought it would be better to know, regardless of what might potentially come out of it. Because then you can prepare yourself, or catch something early… I’ve also had them tested via one DTC-GT company, and I’m waiting for a sale from another company and then I’ll also have their DNA tested there, because they’re testing for very different markers.’ (P12, F42, HL+, NUM+, l.88 and l448)* | Q125 |
|  | *‘Well, it has played through my head, but they’re still rather young, they’re 5 years old now, so maybe later… maybe when they’re a bit older, like ten, eleven, twelve, when they’re more aware of it themselves.’ (P15, M41, HL+, NUM+, l.314-322)* | Q126 |
|  | *‘Well, it wasn’t totally honest of me, but that’s how it went. With those little insights, I did already do that for someone else as well. I did tell them I had uploaded the data to several providers, but not that I did that for these insights. [Interviewer: ‘So if I understand correctly, you unlocked these results without discussing that with the person in question?’] Yes.’ (P14, F38, HL+, NUM-, l.286-292)* | Q127 |
|  | *‘For example, we discovered for someone that they had a high risk for all sorts of auto-immune diseases, so I thought: ‘Well, that isn’t fun for them to know.’, so I didn’t tell that to that person, just about the stuff they already were suffering from… I know this person already has a certain auto-immune disease, and for others they also scored as ‘high-risk’. So I thought: ‘Should I tell this person or not?’. They’re quickly frightened, so I didn’t do that… But yeah, I do know it, so now I’m stuck with it. That’s not very nice.’ (P14, F38, HL+, NUM-, l.36 and l.84-88)* | Q128 |
| Satisfaction with DTC-GT consumer journey | *‘Well, definitely a 9… I’m fully satisfied. Due to this test I’ve gotten what I wanted.’ (P1, F59, HL+, NUM-, l.964-966)* | Q129 |
|  | *‘I might do some searching online to see if there’s people that did multiple tests and multiple companies and compare the results. I know there’s a person on YouTube that did like 6 different DTC-GTs and compared the results of those for example.’ (P2, M70, HL+, NUM+, l.700)* | Q130 |
| Suggestions by consumers to enhance the DTC-GT consumer journey | *‘Yeah, no, look, in hindsight, I think it’s important that people are informed about potential consequences that doing such a test can have. I mean, it’s not nothing… Imagine that you discover you’ve got the BRCA-gene for breast cancer or something… I think it would have a lot of impact if you discover that… I’m not sure if I would’ve done the test if I had known in advance that that would be possible… Yeah, I think it would be handy if the seller gives some type of disclaimer or something.’ (P12, F42, HL+, NUM+, l.130-134 and l.152)* | Q131 |
|  | *‘Uh, yeah, I basically had to gather those bits of information myself, stuff like ‘What am I supposed to pay attention to?’. I think I got that from some article like: ‘What’s the points, quality requirements that I have to pay attention to when selecting a seller?, that was in an American scientific article or something. It would be handy if there was a website for that like: ‘If you’re considering undergoing DTC-GT, pay attention to this and that and that.’, and what also would’ve been handy was a list with what certain terms mean… It doesn’t matter who makes it [the website], as long as it’s made… It has to be good, reliable, maybe the Dutch Institute for Public Health and the Environment… Hmmm, and isn’t there an association of Dutch clinical geneticists {VKGN]? I think that would fit well.’ (P6, F61, HL+, NUM+, l.920-932)* | Q132 |
|  | *‘Well I think, if you have questions about it and want that, you have to be able to reach the party that offers the test. If you can’t get the required information via Internet or a brochure, if you want to ask questions, you have to have a phone number, which is already quite hard these days. Of course you’ll get one-sided information that way, but it would be nice. At least you have a phone number then, and someone you can call.’ (P9, M50, HL+, NUM-, l.706-708)* | Q133 |
|  | *‘Uh, well, also being able to communicate in Dutch. Well the results, because the communication… Yeah, the communication and the results. You get an email once in a while, but that basically says nothing. Uh, but at least having the results in Dutch, I think that would be beneficial.’ (P4, M64, HL+, NUM+, l.642-644)* | Q134 |
|  | *‘Uh, well, I found that part about the enzymes, look everyone knows what colon cancer is, those were clear-cut definitions. But with the enzyme part, you get ‘QZM and I-don’t-know-what’ enzymes and them I’m thinking: ‘I don’t know what that means.’, and then you can open it and read a bit more about that the enzyme plays a role in digestion or whatever, but that was a bit of ‘abracadabra’ sometimes, making me think: ‘Whatever.’… I think that was slightly hard to understand.’ (P8, F40, HL+, NUM-, l.214-218)* | Q135 |
|  | *‘Yeah, something from the seller like ‘…And always discuss these results with an expert, with a specialist.’… ‘If you still have questions or if something bothers you or something made you scared, please contact…’ and then I don’t know to what degree you can expect that from a seller but you know, I’m not sure with whom you should [make contact], whether that’s your GP, or the seller, or a specialist.’ (P5, M54, HL+, NUM-, l.582-588)* | Q136 |
|  | *‘Yeah, I think specifically for people that discover they carry that breast cancer gene, yeah, that there should be somewhere within the public healthcare system where you can go, that you know you can go there. Imagine you get such a result, where should you go? I don’t know.’ (P12, F42, HL+, NUM+, l.186)* | Q137 |

*a: Legend: (participant, gender and age, health literacy high/low, numeracy high/low, lines from respective transcript from which quote was taken). Example Q1: (P3, F42, HL+, NUM-, l.80): quote by participant 3, a female aged 42 with high health literacy and low numeracy, taken from transcript line 80. Example Q63: (P17, M52, HL-, NUM+, l.83-94): quote by participant 17, a male aged 52 with low health literacy and high numeracy, taken from transcript lines 83-94.*
